# Supplementary material for: Understanding Reproductive Health among Survivors of Paediatric and Young adults (URHSPY) cancers in Uganda: A mixed method study protocol
Source: PLoS One. 2023 Apr 25;18(4):e0284969. doi: 10.1371/journal.pone.0284969 (PMC10128918; doi:10.1371/journal.pone.0284969)
Supplement: S1 File — (DOCX) [file pone.0284969.s001.docx]

# Interview Discussion Guide for Health workers

***Opening:*** *My name is [interviewer’s name]. In this study, we are trying to understand the barriers and enablers to addressing reproductive health problems that occur because of cancer treatment in children and young adults with cancer. I would like to ask you some questions about your medical background, your knowledge and your experiences working with children and adolescents with cancer. The interview will take about* ***45 minutes****. You do not have to take part if you do not want to. Please feel free to ask for any breaks that you might need during the interview. This interview will be audio recorded and your identity will remain anonymous. The interview can stop at any time if you want, without risking any penalty. We hope to use this information to help advocate for protection of future fertility among children and young adults diagnosed with cancer.*

*Do you have any questions about the study or interview before I begin? Y/N*

Understanding factors surrounding addressing reproductive health problems resulting from cancer treatment in children and young adults.

**Transition:** Now, I will start by asking you some questions about your profession and your work experience.

**STAGE 1: Introduction or Icebreaker**

1. What is your role at the Uganda Cancer Institute?

Icebreaker, reinforces rapport and gives context for later questions

Understand what s/he knows about the delayed effects of cancer treatment. Understand the effect on some cancer treatment options on future fertility, what does h/she think about this. Understand preparation parents and children for these effects both physical and psychological preparation

**STAGE III: Long term effects of cancer treatment**

OPTIONAL: Can you describe to me the long-term effects of cancer treatment on a survivor’s general wellbeing?

Probe:

(a) What should the child and his or her parent/caretaker expect in terms of long-term effects of cancer treatment?

(b) What are your major worries in terms of complications?

(c) How is the plan to follow up for these long-term effects?

(d) How are childbearing expectations of survivors managed during care and during follow up?

(e) How do you think these expectations are of importance?

Understand the enrolment process. Understand the information given, their understanding of any side effects, understand any issues related to the child’s future fertility, understand information given to parent and or child and their involvement in decision making especially benefits versus risks to future fertility?

**STAGE II: Enrolment into cancer care**

1. Take me through the process of enrolling a child into cancer care at the Uganda Cancer Institute.

Probe:

(a) How is the child informed about his/her condition?

(b) To what extent are the parents involved?

(c) What carders are typically involved in enrolment?

(d) What are your typical concerns during this process?

(c) How much information about expected adverse effects is given to the child and or the parent?

(e) How are concerns about future childbearing managed?

(d) What procedures are done to limit these adverse effects?

Understand what s/he knows about the effects of cancer treatment menstrual health, onset of puberty, age at menopause. Understand what value s/he attached to female reproductive function during care. The extent to which the parent or child is informed. Their experience when interacting with parents and children about these effects and their implications.

1. What do you know about the effect of some cancer treatments on menstruation?

Probe:

(a) If yes, how do you think so?

(b) If no, some cancer treatments interfere with the survivor’s ability to have normal menstrual cycles and to some extent some girls develop early menopause. This can result from surgery on ovaries or testis, chemotherapy, or radiotherapy

(c) How important is knowing these effects on survivor’s menstrual cycles and how does it affect care? Is it something you would consider about during care?

(e) How would you value taking steps to limit the effect of some cancer treatment on the survivor’s menstrual cycle?

Understand what s/he knows about the effects of cancer treatment on a child’s future fertility. Understand what value s/he attached to future fertility during providing care to children. Importance of future childbearing as a component of their child’s survival. Understand their experience discussing this with parents and or the child.

**STAGE III: Impact of cancer treatment on future fertility**

1. What do you know about the effect of cancer treatments on a survivor’s ability to have children in the future?

Probe:

(a) If yes, how do you think so?

(b) If no, some cancer treatments interfere with the survivor’s ability to have children these can be surgery on ovaries or testis, chemotherapy, or radiotherapy

(c) How important is knowing these effects on survivor’s childbearing ability and how does it affect care? Is it something you think about during care?

(d) How would you value taking steps to limit the effect of some cancer treatment on the survivor’s ability to have children?

Understand what s/he thinks about fertility preservation in cancer patients at risk. Especially in children and adolescents. Understand the feasibility of adding fertility preservation into care for children and adolescents with cancer.

1. Knowing about the impact of some cancer treatment on a young cancer survivor’s ability to have children, how does that impact your practice?

Probe:

(a) Is fertility preservation something to consider incorporating into routine care?

(b) If so, how should it be addressed and incorporated into routine care?

(c) What may make it easier to incorporate these changes into routine care?

(d) What may make it more difficult to incorporate these changes into routine care?

Understand what s/he knows about fertility preservation in cancer patients at risk. Especially in children and adolescents. Understand what value s/he attached to fertility preservation in the care of children with cancer. Interactions with parents and children concerning fertility preservation.

1. What do you know about fertility preservation in cancer patients?

Probe:

(a) If yes, what do you know about fertility preservation for young cancer patients?

(b) If no, fertility preservation is a set of procedures and treatment aimed at protecting the potential of an individual to have children in the future.

(c) How important is fertility preservation in the care for young cancer patients?

Thank for your participation

Understand the barriers to addressing future fertility and reproductive health in children with cancer at the health facility and patient level

Understand the facilitators to addressing future fertility and reproductive health in children with cancer at the health facility and patient level

1. On the other hand, what makes it difficult for you to consider a young cancer survivor’s ability to have children and general reproductive health after cancer treatment?

Probe:

Are there things (clinic workflows, individuals/personnel, etc.) available at the cancer Institute that may make it challenging for providers to initiate these conversations with young patients?

**STAGE IV: Conclusion**

1. What would make it easier for you to consider a young cancer survivor’s ability to have children and general reproductive health after cancer treatment?

Probe:

Are there things (clinic workflows, individuals/personnel, etc.) available at the cancer Institute that may make it easier for providers to initiate these conversations with young patients?
