# Supplementary material for: Understanding Reproductive Health among Survivors of Paediatric and Young adults (URHSPY) cancers in Uganda: A mixed method study protocol
Source: PLoS One. 2023 Apr 25;18(4):e0284969. doi: 10.1371/journal.pone.0284969 (PMC10128918; doi:10.1371/journal.pone.0284969)
Supplement: S2 File — (DOCX) [file pone.0284969.s002.docx]

# INTERVIEW DISCUSSION GUIDE FOR PARENTS OR CARETAKERS WITH COMMENTARY

# Introduction

My name is [interviewer’s name]. In this study, we are trying to understand the issues that support or prevent addressing reproductive health problems that occur because of cancer treatment in children with cancer. I would like to ask you some questions about your background, your child’s condition, experiences you have had during your child’s treatment, and about you.

This interview will take about 45 minutes, it will be recorded but your identity and that of your child will not be known. You do not have to take part if you do not want to. Please feel free to ask for any breaks that you might need during the interview. We hope to use this information to raise awareness and support the protection of future childbearing ability among children and young adults found with cancer.

Do you have any questions about the study or interview before I begin? Y/N

**STAGE1: Introduction or Icebreaker**

Tell me about your child.

Probes:

(a) How old are they?

(b) Are they school going?

(c) What is their interest?

(d) How old was your child when you received the diagnosis?

(e) How did your child react to the news about his/her diagnosis?

(d) How did you react to this news about your child’s cancer diagnosis?

Commentary

Icebreaker, reinforce rapport and gives context for later questions

**STAGE II: Enrolment into cancer care and cancer treatment**

Take me through your child’s enrolment into cancer care at the Uganda Cancer Institute.

Probes:

(a) What were you told to expect?

(b) Who was involved in your enrolment?

(c) Were concerns about your child’s future childbearing raised during this process? If so, how was it discussed with you and your child?

(d) How was your child involved in the enrolment process?

(e) What were your expectations during enrolment? Follow with what were the expectations of your child?

(e) Did the enrolment meet your expectations? If not, how could it have been improved upon?

Commentary

Understand the enrolment experience of the parent. Understand the information given, their understanding of any side effects, understand any issues related to the child’s future fertility, how their opinion was incorporated into the treatment decision process regarding choice of treatment especially benefits versus risks to future fertility? Incorporation of the child’s opinion. Their treatment expectations.

Tell me about your child’s cancer treatment.

Probes:

(a) Do you know the types of treatment your child has received?

(b) How did the treatments make your child feel?

(c) Did your child’s body go through any unexpected changes?

(d) Additional probes: If yes, what changes did s/he experience?

(e) Were these changes expected?

(f) How were these changes addressed? What information about these changes was shared with you or and your child?

Commentary

Understand what they know about the treatment the child received. Their understanding of the changes if any in the child after starting the treatment, were these changes expected. How prepared were they for these changes? If the child is female and having already started her menses, where there any changes in her menstrual cycle, Understand the interaction with the health worker about these changes.

**STAGE III: Long term effects of cancer treatment**

What do you know about the long-term effects of cancer treatment on your child’s wellbeing.

Probes:

(a) What were you told about the possible long-term effects of cancer treatment on your child’s future ability to have children?

(b)How was this expressed to you?

(c) Did your child express to you at any point during his/her cancer care a desire to have children? How was this shared or expressed to you?

(d) Is future childbearing for your child important to you?

(e) If yes, how is future childbearing for your child important?

(f) Was this concept of future childbearing addressed during your child’s treatment?

(g) additional probes: If not, how would you have preferred to have this concept shared and discussed with you and your child? If yes, how was the issue of childbearing discussed with you? How was your opinion incorporated into the treatment decision? How was your child's the opinion incorporated into the treatment decision?

Commentary

Understand what s/he knows about the delayed effects of cancer treatment, how s/he understands the effect of some cancer treatment options on future fertility, what does h/she think about this. How prepared is s/he and child for these effects? Importance of future childbearing as a component of their child’s survival. Understand their experience discussing this with the health worker.

**STAGE IV: Conclusion**

If you knew about the impact of some cancer treatments on your child’s ability to have children in the future, how would this have changed your cancer treatment experience?

Probe as needed:

(a) What information about the impact of cancer treatment on your child’s future childbearing do you think other parents would need to know as their children start treatment?

(b) Would they need more information to prepare them for what to expect concerning delayed effects of treatment?

(c) What information do you wish you had now about the impact of cancer treatment on your child’s future fertility?

(d) What do you think the health workers caring for children with cancer should do differently when preparing to start treatment?

Commentary

Understand what s/he thinks about the impact of cancer treatment on the child’s future fertility. And if his/her overall cancer treatment experience would be different if h/she were told about this- and in what way.

Understand what kind of decision-making process h/she would have wanted, other information h/she was missing before their child started cancer treatment, whether h/she understood the risks or complications, to future fertility. how h/she could be better served now during the child’s follow up care

**Thank for your participation**

**Notes:**
