# Supplementary material for: Understanding Reproductive Health among Survivors of Paediatric and Young adults (URHSPY) cancers in Uganda: A mixed method study protocol
Source: PLoS One. 2023 Apr 25;18(4):e0284969. doi: 10.1371/journal.pone.0284969 (PMC10128918; doi:10.1371/journal.pone.0284969)
Supplement: S3 File — (ZIP) [file pone.0284969.s003.zip › CRF indepth_oncoteam v1.1 210621.pdf]

Uganda Cancer Institute
Research is Our Resource

CRF: Health workers

v1.1

1. **Consented**

☐Yes
☐No

(IF No, do not proceed)

2. **Site ID**

____

(Default is UCI)

3. **Study ID**

____

4. **Subject ID**

____

5. **Interview date**

____

6. **Age**

____

7. **Gender**

☐Male
☐Female

8. **Home address**

____

9. **Marital status**

☐Married
☐Living with a partner
☐In a committed relationship but not living together
☐Single
☐Other

(Choose one)

10. **Religion**

☐Catholic
☐Anglican
☐Moslem
☐Pentecostal
☐Orthodox
☐Other

(Choose one)

11. Professional category

☐Oncologist
☐Medical officer
☐Registered nurse
☐Senior resident
☐Other

(Choose one)

12. Duration of practice (In oncology discipline)

☐less than 1 year
☐1-5 years
☐5-10 years
☐More than 10 years

13. Interviewer

________________________ __________________

(Initials) Date

14. CRF checked by

________________________ __________________

(Initials) Date
