# Supplementary material for: Understanding Reproductive Health among Survivors of Paediatric and Young adults (URHSPY) cancers in Uganda: A mixed method study protocol
Source: PLoS One. 2023 Apr 25;18(4):e0284969. doi: 10.1371/journal.pone.0284969 (PMC10128918; doi:10.1371/journal.pone.0284969)
Supplement: S3 File — (ZIP) [file pone.0284969.s003.zip › CRF indepth_parent or caretaker v1.2 270621.pdf]

Uganda Cancer Institute
Research is Our Resource

CRF: Parent or caretaker

v1.2

1. **Consented**

☐Yes
☐No

(IF No, do not proceed)

2. **Site ID**

____

(Default is UCI)

3. **Study ID**

____

4. **Subject ID**

____

5. **Interview date**

____

6. **Age**

____

7. **Gender**

☐Male
☐Female

8. **Home address**

____

9. **Relationship to the child**

☐Parent
☐Uncle or auntie
☐Grandparent
☐Other caretaker

(Choose one)

10. **Gender of the child**

☐Male
☐Female

11. **Marital status**

☐Married
☐Living with a partner
☐In a committed relationship but not living together
☐Single
☐Other

(Choose one)

12. Religion

☐Catholic
☐Anglican
☐Moslem
☐Pentecostal
☐Orthodox
☐Other

(Choose one)

13. Education background

☐Less than primary school
☐Primary level graduate
☐Lower secondary level graduate
☐Higher secondary level graduate
☐Diploma level
☐Degree level
☐Postgraduate level

(Choose one)

14. Tribe

☐Baganda
☐Banyakole
☐Basoga
☐Bakiga
☐Itesot
☐Langi
☐Bagishu
☐Acholi
☐Lugbara
☐Other

(Choose one)

15. Child's cancer diagnosis

____

16. Child's cancer treatment

☐Surgery
☐Radiotherapy
☐Chemotherapy
☐Hormonal treatment
☐Other
☐Unknown

(Tick multiple if applicable)

17. Interviewer

________________________ (Initials) ____________________ Date

18. CRF checked by

________________________ (Initials) ____________________ Date
