# Supplementary material for: Understanding Reproductive Health among Survivors of Paediatric and Young adults (URHSPY) cancers in Uganda: A mixed method study protocol
Source: PLoS One. 2023 Apr 25;18(4):e0284969. doi: 10.1371/journal.pone.0284969 (PMC10128918; doi:10.1371/journal.pone.0284969)
Supplement: S3 File — (ZIP) [file pone.0284969.s003.zip › URHSPY CATI_female v2.1 210621.pdf]

Uganda Cancer Institute
Research is Our Resource

A Population-Based survey of reproductive health among survivors of pediatric and young adult cancers in Uganda

Computer Assisted Telephone Interview (CATI)

FEMALE VERSION

Principal Investigator: Anthony Kayiira
Co-investigators: Joyce Kambugu, Daniel Zaake, Serena, Henry Wabinga, Rahel Ghebre

Sponsor: NPGH Consortium,
Fogarty International Centre,
National Institutes of Health,
National Institutes of Health's National Center for Advancing Translational Sciences

Date: Sunday, June 27, 2021

VERSION 2.1

Confidential. Do not distribute without permission.
