# Supplementary material for: Understanding Reproductive Health among Survivors of Paediatric and Young adults (URHSPY) cancers in Uganda: A mixed method study protocol
Source: PLoS One. 2023 Apr 25;18(4):e0284969. doi: 10.1371/journal.pone.0284969 (PMC10128918; doi:10.1371/journal.pone.0284969)
Supplement: S3 File — (ZIP) [file pone.0284969.s003.zip › URHSPY CATI_male v2.0 210621.pdf]

A Population-Based survey of reproductive health among survivors of pediatric and young adult cancers in Uganda

Computer Assisted Telephone Interview (CATI)

MALE VERSION

Principal Investigator: Anthony Kayiira
Co-investigators: Joyce Kambugu, Daniel Zaake, Serena, Henry Wabinga, Rahel Ghebre

Sponsor: NPGH Consortium,
Fogarty International Centre,
National Institutes of Health,
National Institutes of Health's National Center for Advancing Translational Sciences

Date: Sunday, June 27, 2021

VERSION 2.1

CONFIDENTIAL. DO NOT DISTRIBUTE WITHOUT PERMISSION.

TABLE OF CONTENTS

TABLE OF CONTENTS 2
SECTION A: PRELIMINARY INFORMATION AND INTRODUCTION TO THE STUDY 3
SECTION B: CANCER HISTORY 4
SECTION C: DEMOGRAPHIC QUESTIONS 10
General Demographic Questions 10
Relationship History 10
Ethnicity and address 11
Children Raised 12
SECTION D: DESIRE FOR CHILDREN 13
Reproductive Goals 14
Reproductive Expectations 14
Importance of Biological Children 15
Feelings about Reproductive Experiences 16
SECTION E: INFERTILITY HISTORY 18
Infertile Period 18
Fertility Preservation 20
Medical Care for Infertility 22
Fertility Tests 24
Infertility Treatments 25
Surgery 26
Medications 27
Insemination 27
Sperm use – Introduction 28
Sperm use– In Vitro Fertilization 28
Sperm use – Fertility Preservation 30
Donor sperms 34
SECTION F: PREGNANCY HISTORY 35
SECTION G: LIFESTYLE 37
Smoking History 37
Alcohol Consumption 42
Income 42
SECTION H: CONCLUSION & CONTACT INFORMATION 43
SECTION I: INTERVIEWER IMPRESSION 44

Confidential. Do not distribute without permission.

SECTION A: PRELIMINARY INFORMATION AND INTRODUCTION TO THE STUDY

ENTER STUDY ID#

ENTER TODAY'S DATE: (MM/DD/YY)

Start of interview time: (HH:MM)

A1. What is your birth date?

_/_ _/_ _ _ _ (mm/dd/yyyy)

NOTE TO INTERVIEWER – USE BIRTHDATE TO CALCULATE CURRENT AGE: CURRENT AGE=TODAY DATE-BIRTHDATE

AGE: ____________ [ENTER TEXT]

In this interview, we'll be discussing a number of topics including your medical and fertility history in addition to information on lifestyle habits that could affect your health. We understand that some things we ask may be difficult to remember. The dates and ages we ask for in many of the questions are important to help us assess changes in your health. Please take the time you need to give us your best estimate of any dates or ages that we ask about. Thank you for your time and patience with this. At the end of the interview, an airtime token worth $3 will be sent to a telecom carrier of your choice.

A2. Have you ever been **treated** for cancer?

No	0	GO TO A8
Yes	1	GO TO A3

A3. IF IN A2=1 (YES): What type of tumor or cancer were you **first diagnosed** with?

Cancer type: [DROP DOWN LIST OF CANCER, PRIMARY 1-22, OTHER AND NONE]
IF OTHER – INSERT IN TEXT BOX THEN GO TO A4
IF NONE – GO TO A6

Confidential. Do not distribute without permission.

A4. IF IN A2=1 (YES) and IF A3= 1-22 OR OTHER: How old were you at the time of diagnosis? [NOTE TO INTERVIEWER– IF MAN REPORTS A RANGE OF AGES, ASK FOR HIS BEST GUESS. PROMPT FOR MONTH AND YEAR IF CANNOT REMEMBER AGE. IF PROVIDE MONTH AND YEAR, CALCULATE AGE.]

Age: ____
Month: ____
Year: ____

[IF MONTH/YEAR, ASK FOR CURRENT AGE AND CALCULATE AGE AT DIAGNOSIS]

IF ANS A4 ≥ 0 OR A4 ≤ 25, GO TO INSTRUCTIONS BEFORE B1

IF ANS A4 ≥ 26, GO TO A5

A5. IF ANS A4 ≥ 26, Thank you for your interest in our study. Unfortunately, we are only recruiting men who were between 1 and 25 when they were first diagnosed with, so you are not eligible. We appreciate your time. Goodbye. [END CALL]

A6. IF A3= NONE, READ: Thank you for your interest in our study. Unfortunately, we are only recruiting men who were diagnosed with cancer. We appreciate your time. Goodbye. [END CALL]

A7. IF ANS A3 ≠ NONE OR ANS A2=1, YES READ: In what HEALTH FACILITY where you diagnosed?

Drop down: Health facilities that contribute to the Kampala Cancer Registry

A8. Thank you for your interest in our study. Unfortunately, at this time we are recruiting men who have been diagnosed with cancer. We appreciate your time. Goodbye. [END CALL]

SECTION B: CANCER HISTORY

I'd like to ask you a few questions about your medical history. Although we will be getting more information about your cancer treatments from the cancer registry, we need to collect some data now.

B1. Who was your primary oncologist or doctor primarily responsible for your cancer care when you were first diagnosed with cancer?

Oncologist name: ____

Confidential. Do not distribute without permission.

B2. What was the name and location of the hospital or clinic where your oncologist or primary doctor worked?
[IF MAN ONLY GIVES NAME, PROMPT: What County and district was this hospital or clinic located?]

Hospital/Clinic name: ____
County or district of hospital/clinic: ____

B3. How were you treated for ***this*** cancer diagnosis? Please say yes or no for each of the following: [NOTE TO INTERVIEWER – COMPLEMENTARY AND ALTERNATIVE TREATMENTS SUCH AS HERBAL THERAPY SHOULD BE AN 'OTHER' CHOICE; READ ALL ANSWER CHOICES]

Radiation	1	GO TO B4
Chemotherapy	2	GO TO B4
Surgery, not including biopsies	3	GO TO B4
Hormone treatment, including Tamoxifen	4	GO TO B4
Another treatment	5	GO TO B4
[DON'T READ]		
Don't remember/don't know	99	GO TO B4

B4. Did you and or your guardian talk to a doctor or other health professional about how this cancer treatment could affect your ability to make a woman pregnant?

No	0	SKIP TO B15
Yes	1	
Don't remember	2	SKIP TO B15

B5. IF B4=1, YES, READ: Did this discussion occur…?

Before treatment	1
During treatment	2
After treatment	3

B6. IF B4=1, YES, Who initiated this discussion? [NOTE TO INTERVIEWER – IF MULTIPLE REPLY "Who *first* brought up the effect of this treatment on your fertility"]

You	1	
Your guardian	2	
Your partner	3	
Your oncologist	4	
Your general practitioner	5	
Your urologist	6	
Your fertility specialist	7	SKIP TO B8

Confidential. Do not distribute without permission.

Another specialist(s) or doctor 8

B7. At that time were you and or your guardian referred to a fertility specialist to discuss your future fertility?

No 0 SKIP TO B15
Yes 1

B8. IF ANS B7=1, YES OR B6=7 (fertility specialist), READ: When were you and or your guardian referred?

Before treatment 1 SKIP TO B9
During treatment 2 SKIP TO B10
After treatment 3 SKIP TO B10

B9. IF ANS B8=1, BEFORE TREATMENT, READ: How long before the start of your cancer treatment were you referred? [NOTE TO INTERVIEWER- IF MAN REPORTS A RANGE OF TIME, ASK FOR HER BEST GUESS. IF MAN SAYS SHE DOES NOT REMEMBER PROMPT HER TO TRY AND ESTIMATE HOW LONG.] SKIP TO B10

Days: ____
Weeks: ____
Months: ____
Don't remember. 99

B10. At that time, did you and or your guardian discuss specific fertility preservation options? Fertility preservation can include taking medications for freezing of sperms or embryos. Sperm freezing is a method used to save men's ability to have children in the future. Sperms are harvested from semen are frozen and stored for later use.
Embryo freezing involves in vitro fertilization, a procedure in which eggs are removed from your partner's ovary and combined with your sperm in the laboratory to form embryos. The embryos are frozen and can later be thawed and placed in your partner's uterus. Embryo freezing is a type of fertility preservation.

No 0 SKIP TO B15
Yes 1

B11. IF ANS B10=1, YES, READ: What fertility preservation methods did you and or your guardian discuss? Please say yes or no for each of the following.

Sperm freezing 1
Testicular tissue freezing 2
Other 3

B12. Did you **use** any fertility preservation methods **before** your treatment for *this cancer* diagnosis?

No 0 SKIP TO B14
Yes 1

Confidential. Do not distribute without permission.

B13. IF ANS B12=1, YES, READ: Which types did you use? Please say yes or no for each of the following.

Sperm freezing	1
Testicular tissue freezing	2
Other	3

SKIP TO B15

B14. IF ANS B12= 0, NO READ: What are some of the reasons you and or your guardian considered not use fertility preservation prior to your treatment for this cancer diagnosis? Please tell me all that apply.

Did not want to delay cancer treatment	1
Concerned about my mortality	2
Did not believe my cancer treatment would affect my fertility	3
Did not want children or did not want more children	4
It was too expensive	5
Options were too overwhelming	6
Did not like available options	7
Decided to wait and reevaluate the situation after cancer treatment	8
Other	9

[DO NOT READ]
Don't know 99

B15. Did a health professional tell you how long you had to wait after completion of your cancer treatment before attempting to get a woman pregnant?

No	0	SKIP TO INSTRUCTIONS BEFORE B17
Yes	1	

B16. IF ANS B15= 1, YES, READ: How long were you told to wait? [NOTE TO INTERVIEWER- IF MAN REPORTS A RANGE OF TIME, ASK FOR HIS BEST GUESS.]

Months: ____
Years: ____

[DO NOT READ]
Don't remember 99

INSTRUCTIONS BEFORE B17: Thinking about health information you or your family may have wanted about your fertility and your fertility preservation options prior to your treatment for this cancer diagnosis, please say whether you strongly agree, agree, neither agree nor disagree, disagree, or strongly disagree with the following statements *for this diagnosis.*:

B17. I received enough information about possible effects of cancer treatment on my future fertility from a medical professional prior to treatment:

Confidential. Do not distribute without permission.

Strongly agree	1
Agree	2
Neither agree nor disagree	3
Disagree	4
Strongly disagree	5

B18. IF ANS B10=1, YES, READ: I felt that my fertility preservation options were well explained:

Strongly agree	1
Agree	2
Neither agree nor disagree	3
Disagree	4
Strongly disagree	5

B19. IF B10=0, NO, READ: I wish a medical professional had discussed fertility preservation prior to my cancer treatment:

Strongly agree	1
Agree	2
Neither agree nor disagree	3
Disagree	4
Strongly disagree	5

B20. Preparing to undergo treatment for cancer was too stressful for me at the time to consider how my treatment would affect my fertility

Strongly agree	1
Agree	2
Neither agree nor disagree	3
Disagree	4
Strongly disagree	5

INSTRUCTIONS BEFORE B21: Now I would like to ask you about recurrences and other cancer diagnoses that you may have had.

B21. Did this same cancer come back again?

No	0	SKIP TO B23
Yes	1	

Confidential. Do not distribute without permission.

B22. IF B21=1, YES, READ: How old were you when you had a recurrence of ***this*** cancer?
[NOTE TO INTERVIEWER- IF MAN REPORTS A RANGE OF AGES, ASK FOR HER BEST GUESS. PROMPT FOR MONTH AND YEAR IF CANNOT REMEMBER AGE]

Age: ____
Month: ____
Year: ____
SKIP TO B26

B23. Have you been diagnosed with any other types of cancer?

No	0	SKIP TO INSTRUCTIONS BEFORE C1
Yes	1	

B24. IF B23=1, YES: What type of cancer were you diagnosed with?

Cancer type: [DROP DOWN LIST OF CANCER]

B25. IF B23=1, YES & B24 NE NONE : How old were you when you were diagnosed with this cancer?
[NOTE TO INTERVIEWER- IF MAN REPORTS A RANGE OF AGES, ASK FOR HER BEST GUESS. PROMPT FOR MONTH AND YEAR IF CANNOT REMEMBER AGE]

Age: ____
Month: ____
Year: ____

B26. IF B21=1, YES OR IF B23=1, YES, READ: Was [AUTOFILL MOST RECENT ONCOLOGIST FROM ANS B1] still your primary oncologist or doctor primarily responsible for your care for *this* diagnosis?

No	0
Yes	1

RETURN TO B3 – B20

B27. IF B21=1, YES OR IF B23=1, YES AND IF B26=0, NO, READ: Who was your primary oncologist or doctor responsible for your care for this cancer diagnosis?

Oncologist or doctor's name: ____

B28. IF B21=1, YES OR IF B23=1, YES What was the name and location of the hospital or clinic where your primary oncologist or doctor worked?
[IF MAN ONLY GIVES NAME, PROMPT: What County and district was this hospital or clinic located?]

Hospital/Clinic name: ____
County or district of hospital/clinic: ____

RETURN TO B3 – B20

Confidential. Do not distribute without permission.

Uganda Cancer Institute
Research is Our Resource

SECTION C: DEMOGRAPHIC QUESTIONS

Now, I'd like to ask you a few questions about how you would describe yourself.

General Demographic Questions

C1. Which of the following best describes your current level of education?

Less than primary school	1
Primary level graduate	2
Lower secondary level graduate	3
Higher secondary level graduate	4
Technical or Vocational school graduate	5
Graduate degree	6
Post graduate degree	7
DO NOT READ	
[REFUSAL]	99

Relationship History

C2. Which of the following best describes your *current* relationship status? [NOTE TO INTERVIEWER – READ ENTIRE LIST, BUT DO NOT READ 'OTHER' OPTION. IF RESPONDENT SAYS NONE OF THE OPTIONS APPLY, CHOOSE OTHER.]

Married	1	
Living with a partner	2	
In a committed relationship but not living together	3	SKIP TO C4
Single	4	SKIP TO C4
[DO NOT READ]		
Other	5	SKIP TO C4

C3. IF ANS C2=1 OR IF ANS C2=2, MARRIED OR LIVING WITH A PARTNER, READ: How many years have you been living together, [including any time prior to your marriage], that you lived together?

[NOTE TO INTERVIEWER – IF LESS THAN 1 YEAR, ENTER 0]

Years	Drop down 0-27
Months	Drop down 1-12

C4. IF ANS C2=1, MARRIED; OR C2=2, LIVING WITH A PARTNER, READ: Prior to this relationship, have you ever married or lived with anyone else as a couple for at least a year?

No	0	SKIP TO C7
Yes	1	

Confidential. Do not distribute without permission.

IF ANS C2=3, IN A COMMITTED RELATIONSHIP BUT NOT LIVING TOGETHER; C2=4, SINGLE; OR C2=5, OTHER, READ: Have you ever married or lived with anyone as a couple for at least a year?

No	0	SKIP TO C7
Yes	1	

C5. IF ANS C4=1, YES, READ: How old were you when you *first* began living with someone as a couple? [NOTE TO INTERVIEWER- IF MAN REPORTS A RANGE OF AGES, ASK FOR HER BEST GUESS. PROMPT FOR MONTH AND YEAR IF CANNOT REMEMBER AGE]

Age: ____
Month: ____
Year: ____

C6. How long did you live with that person? [NOTE TO INTERVIEWER – IF A MAN REPORTS A RANGE OF YEARS, PROMPT FOR BEST GUESS]

Years Drop down 0-27
[NOTE TO INTERVIEWER – IF LESS THAN 1 YEAR, ENTER 0]

Ethnicity and address

Now I'm going to ask you a few questions about your ethnicity and where you live

C7. Which of the following would you say best describes your tribe? Please indicate all that apply.

Baganda	1
Banyakole	2
Basoga	3
Bakiga	4
Itesot	5
Langi	6
Bagishu	7
Acholi	8
Lugbara	9
Other	10
[DO NOT READ]	
REFUSAL	99

Confidential. Do not distribute without permission.

C8. Do you reside in Uganda?

No	0 SKIP TO C10
Yes	1

C9. IF 0=1, YES In which sub region do you reside?

Drop down list of sub regions: 10

C10. IF 0=0, NO: What country do you reside?

Text box: ENTER VALUE

C11. IF 0=1: Which location category best describes where you currently live?

Urban	1
Rural	2

Children Raised

INSTRUCTIONS BEFORE C12: Now I would like to ask you some preliminary questions about your reproductive and family history. We are interested in children who are biologically related to you as well as children you may have adopted or raised. We will come back to some of these topics later on.

C12. How many children have you fathered? Please count any child you fathered regardless of whether you raised the child or not. Please do not include any children born as still births at this time.

Number: ____ IF ANS>0, SKIP TO C16

C13. IF ANS C12= 0, READ: Have you ever tried to have a child?

No	0
Yes	1

C14. Have you raised any children? Include any children you feel you played a significant role in raising or who may have passed away during infancy or childhood. This may include children who you were not legally responsible for or children who spent part of their time in another household.

No	0	SKIP TO INSTRUCTIONS BEFORE D1
Yes	1	

C15. IF ANS C14=1, YES, READ: How many?

Confidential. Do not distribute without permission.

Uganda Cancer Institute
Research is Our Resource

Number: ____

C16. Did you raise any children that you did not father; including children you adopted, stepchildren or other children who you feel you played a significant role in raising?

No	0	SKIP TO INSTRUCTIONS BEFORE D1
Yes	1	

C17. IF ANS C16=1, YES, READ: How many?

Number: ____

C18. IF ANS C16=1, YES OR IF ANS C14=1, YES How many of these children are children you adopted?

Number of adopted children: ____

C19. IF C18>0: Which of the following best describes the reasons you decided to adopt a child? Please tell me all that apply.

I was unable to have as many biological children as I wanted	1
I thought it would be socially responsible to adopt	2
I wanted a child but had no partner	3
I adopted my partner's child or children	4
I adopted a child or children of a friend or family member who couldn't raise them	5
Another reason	6

C20. IF ANS C16=1, YES OR IF ANS C14=1, YES How many of the children you have raised are stepchildren or children of your partner?

Number of children: ____

SECTION D: DESIRE FOR CHILDREN

INSTRUCTIONS BEFORE D1: 1Some men want to have children, and some don't. Some men are able to have the family size they want, and others are not. We are interested in learning more about how you feel about children. We will use this information to compare your desire for children before and after your cancer treatment.

Confidential. Do not distribute without permission.

Uganda Cancer Institute
Research is Our Resource

Reproductive Goals

D1. Ideally, how many children would you like to raise? Include children you already have as well as children you would like to have in the future. [NOTE – IF MAN PROVIDES A RANGE, PLEASE MARK THE LOWER AND UPPER NUMBER]

Number of children: ____

[DO NOT READ]
Lower number: ________
Upper number: ________

Never thought about it	90
Don't know	99

IF [ANS D1] > 0 CHILDREN OR [ANS D1] > 0 LOWER NUMBER OF CHILDREN SKIP TO D3

D2. IF [ANS D1] = 0 CHILDREN OR [ANS D1] = 0 LOWER NUMBER OF CHILDREN READ: Men have different reasons for not wanting to have children. I am going to read you a list of some common reasons' men decide not to have children. Please tell me each one that applies to you.

Prefer lifestyle without children	1
Not ready for children	2
Don't enjoy children	3
Too expensive	4
Worried about raising a child by myself	5
Partner does not want a child or more children	6
Worried about being a good parent	7
Worried about being too old	8
Worried about not being able to make a woman pregnant	9
Uncomfortable with the idea of using in vitro fertilization or other Medical methods to help me get child	10
Worried won't live long enough to raise a child	11
Medical Health reasons	12
Another reason	13

Reproductive Expectations

D3. Which of the following best describes your expectations about the number of children you will raise? [NOTE TO INTERVIEWER: READ ALL OPTIONS; IF THEY WANT 0 CHILDREN AND EXPECT TO HAVE 0 CHILDREN, CHOOSE 1]

I will probably raise the number of children that I want	1	SKIP TO INSTRUCTIONS BEFORE D6
I will probably raise fewer children than I want	2	
I will probably raise more children than I want	3	SKIP TO D5

Confidential. Do not distribute without permission.

D4. IF [ANS D5] = 2 Why do you think you will raise fewer children than you want? Please tell me each one that applies to you:

Don't have enough time	1
Can't afford that many children	2
No partner	3
My partner does not want that many children	4
Concerned about my age	5
Unable to get a woman pregnant	6
Concerned about my ability to get a woman pregnant	7
Don't want to use in vitro fertilization or other medical methods to help me get a child	8
Don't want to adopt	9
Don't think I can adopt	10
Health reasons	11
Worried about the baby's health	12
Worried about living long enough to raise my children	13
Another reason	14

IF [ANS D3] = 2 SKIP TO INSTRUCTIONS BEFORE D6

D5. IF [ANS D3] = 3 Why do you think you might later raise more children than you currently want? Please tell me each one that applies to you:

My views might change when I reach a different stage in my life	1
Being in a relationship might change my mind	2
My financial status might improve	3
My lifestyle might change	4
My partner or I might want a child of a specific sex	5
My partner or I might want a sibling for our child or children	6
My partner or I might want more children	7
I might get a woman pregnant and decide I want the child	8
I might get involved with someone who already has children	9
Another reason	10

Importance of Biological Children

D6. When you were 18, how important was it that you have children that were related to you biologically at some point?

Very important	1
Somewhat important	2
Neither important nor unimportant	3
Somewhat unimportant	4
Very unimportant	5

Confidential. Do not distribute without permission.

D7. Currently, how important is it to you that you have at least one child that is related to you biologically? That child can be one you already have or one you want to have in the future.

Very important	1
Somewhat important	2
Neither important nor unimportant	3
Somewhat unimportant	4
Very unimportant	5

Feelings about Reproductive Experiences

Now we want to learn more about how you feel about having a, or another biological child. Some men feel uncomfortable talking about how they feel about having children. We are asking questions about this so we can better understand how men feel.
For each of the following statements, please say whether you strongly agree, agree, neither agree nor disagree, disagree, or strongly disagree. [NOTE TO INTERVIEWER, IF ANSWERS 'SOMEWHAT AGREE/DISAGREE,' PROBE FOR WHETHER SHE 'STRONGLY AGREES/DISAGREES' OR 'AGREES/DISAGREES.' IF CANNOT DECIDE, CHOOSE AGREE/DISAGREE.]

D8. I want to have a biological child or another biological child [NOTE: IF MAN SAYS HE CANNOT HAVE ANOTHER CHILD PICK NOT APPLICABLE AND SKIP TO D13]

Strongly agree	1	
Agree	2	
Neither agree nor disagree	3	
Disagree	4	
Strongly disagree	5	
DO NOT READ		
Not Applicable	98	SKIP TO D13

D9. I would be disappointed if I found out I could not get a woman pregnant

Strongly agree	1
Agree	2
Neither agree nor disagree	3
Disagree	4
Strongly disagree	5

Confidential. Do not distribute without permission.

D10. I would be comfortable with the idea of using medically assisted reproduction, such as in vitro fertilization or artificial insemination, to help me get a child
[IF MAN SAYS HE DOES NOT NEED MEDICALLY ASSISTED REPRODUCTION, ASK HIM HOW HE WOULD FEEL IF HYPOTHETICALLY HE DID NEED MEDICALLY ASSISTED REPRODUCTION]

Strongly agree	1
Agree	2
Neither agree nor disagree	3
Disagree	4
Strongly disagree	5

D11. I would be comfortable with the idea of adopting a child

Strongly agree	1
Agree	2
Neither agree nor disagree	3
Disagree	4
Strongly disagree	5

D12. I will be satisfied with my life if I am unable to have a child or more children.

Strongly agree	1
Agree	2
Neither agree nor disagree	3
Disagree	4
Strongly disagree	5

IF D8 NE 98 NOT APPLICABLE, SKIP TO INSTRUCTIONS BEFORE E1

D13. IF D8 = 98 NOT APPLICABLE READ: Had circumstances allowed, I would have liked to have a biological child or more biologicalal children

Strongly agree	1
Agree	2
Neither agree nor disagree	3
Disagree	4
Strongly disagree	5

D14. IF IF D8 = 98 NOT APPLICABLE READ I was disappointed when I found out I could not get pregnant a woman pregnant

Strongly agree	1
Agree	2
Neither agree nor disagree	3
Disagree	4
Strongly disagree	5

Confidential. Do not distribute without permission.

D15. IF D8 = 98 NOT APPLICABLE READ I would be comfortable with the idea of using medically assisted reproduction, such as in vitro fertilization or artificial insemination, to help me get a woman pregnant

Strongly agree	1
Agree	2
Neither agree nor disagree	3
Disagree	4
Strongly disagree	5

D16. IF D8 = 98 NOT APPLICABLE READ I would be comfortable with the idea of adopting a child

Strongly agree	1
Agree	2
Neither agree nor disagree	3
Disagree	4
Strongly disagree	5

D17. IF D8 = 98 NOT APPLICABLE READ I will be satisfied with my life, despite the fact that I am unable to have a child or more children

Strongly agree	1
Agree	2
Neither agree nor disagree	3
Disagree	4
Strongly disagree	5

SECTION E: INFERTILITY HISTORY

INSTRUCTIONS BEFORE E1: Now, I want to ask you some questions about your fertility over the years. The following questions will ask you about various dates and ages related to your fertility. We will use this information to compare your fertility before and after your cancer treatment.

E1. Have you ever been told by a medical professional that you have a medical condition that could prevent you from making a woman pregnant?

No	0
Yes	1

Infertile Period

Some men get a woman pregnant the first time they have sex if she and him are not using birth control, but other men may take a long time to get a woman pregnant or not get a woman pregnant even though they are having sex regularly and they are not using birth control.

Confidential. Do not distribute without permission.

We are interested in times when a man does not get a woman pregnant even though they are having regular intercourse and they aren't doing anything to prevent pregnancy. The following questions do not refer to a time after vasectomy.

E2. Have you ever had sexual intercourse with a female partner?

No	0	SKIP TO E9
Yes	1	

E3. Has there ever been a period of time during which you had unprotected sex with a female partner for 12 months or longer, but you did not get her pregnant? Only count periods of time when you had sex at least once a week. [NOTE TO INTERVIEWER, IF ASKED, IF THE MAN GETS A WOMAN PREGNANT IN MONTH 12 OR LATER, BUT STILL HAD ANOTHER 12 MONTHS WITHOUT GETTING HER PREGNANT, THEN ANSWER IS 'YES']

No	0	SKIP TO E9
Yes	1	

E4. How old were you at the beginning of this time period? It may help to think about who your partner was at the time. [NOTE TO INTERVIEWER- IF MAN REPORTS A RANGE OF AGES, ASK FOR HER BEST GUESS. PROMPT FOR MONTH AND YEAR IF CANNOT REMEMBER AGE]

Age: ____

[IF CANNOT REMEMBER AGE, PROMPT FOR MONTH AND YEAR]
Month: ____
Year: ____

E5. Were you actively trying to get your partner pregnant at this time?

No	0
Yes	1

E6. For how many months or years did this continue? [NOTE TO INTERVIEWER – IF ANSWER IS LESS THAN 6 MONTHS, PROMPT FOR TOTAL TIME (SHOULD BE AT LEAST 6 MONTHS). IF MAN REPORTS A RANGE OF MONTHS/YEARS, ASK FOR HER BEST GUESS.]

Months: ____
Years: ____

E7. Did you get your partner pregnant at the end of this time period?

No	0
Yes	1

Confidential. Do not distribute without permission.

E8. Was there another time you had unprotected sex for 12 months or longer but didn't get your partner pregnant?

No	0	
Yes	1	RETURN TO E4

Fertility Preservation

E9. IF B10= 1 READ: We already asked you about fertility preservation in the context of your cancer treatment. Now we would like to know about any experiences with fertility preservation, such as freezing your sperms that were unrelated to your cancer treatments. **Sperm freezing is a method used to save men's ability to get a woman pregnant in the future. Sperms harvested from your semen are frozen and stored for later use. Embryo freezing involves in vitro fertilization, a procedure in which eggs are removed from a woman's ovary and combined with your sperm in the laboratory to form embryos. The embryos are frozen and can later be thawed and placed in a woman's uterus. Embryo freezing is a type of fertility preservation.**

Aside from when you were being treated for cancer, have you ever discussed fertility preservation with a medical professional? Do not count procedures related to getting a woman pregnant, such as artificial insemination or in vitro fertilization. We will talk about these procedures later.

No	0	SKIP TO INSTRUCTIONS BEFORE E16
Yes	1	

IF B10= 0 READ: Now, I would like to talk to you about your experiences with fertility preservation. Fertility preservation offers a way for men to preserve their sperm so that they can have children in the *future*. It involves procedures such as sperm or embryo freezing. **Sperm freezing is a method used to save men's ability to get a woman pregnant in the future. Sperms harvested from your semen are frozen and stored for later use. Embryo freezing involves in vitro fertilization, a procedure in which eggs are removed from a woman's ovary and combined with your sperm in the laboratory to form embryos. The embryos are frozen and can later be thawed and placed in a woman's uterus. Embryo freezing is a type of fertility preservation**.

Have you ever discussed fertility preservation with a medical professional? Do not count procedures related to getting a woman pregnant, such as artificial insemination or in vitro fertilization. We will talk about these procedures later.

No	0	SKIP TO INSTRUCTIONS BEFORE E16
Yes	1	

E10. IF E9=1, YES READ: Who did you talk to? Please tell me all that apply.

Confidential. Do not distribute without permission.

General practitioner	1
Urologist	2
Fertility specialist, such as a reproductive endocrinologist	3
Another specialist(s) or doctor	4

E11. IF E9=1, YES. At what ages did you discuss fertility preservation with a medical professional? [NOTE TO INTERVIEWER, PROMPT FOR MONTHS AND YEARS IF CANNOT REMEMBER AGES. IF MAN REPORTS A RANGE OF AGES, PROMPT FOR HER BEST GUESS]

Age1: _____
Age2: _____

Date1: ________ (MM/ YYYY)
Date2: ________ (MM/ YYYY)

E12. Why did you discuss fertility preservation? Please tell me all that apply.

Because you are a cancer survivor

1
Because you had a health condition that might affect your fertility

2
Because you were worried your fertility would decrease before you found a partner

3
Because you were worried it would be harder to get a woman pregnant when you were older

4
Other

5

E13. IF E9=1, YES Did you **USE** any of the fertility preservation methods?

No	0	GO TO E14
Yes	1	SKIP TO E15

E14. IF E13 = 0, NO READ: Why did you choose not to have fertility preservation done? Please tell me all that apply.

Tried to get a woman pregnant immediately	1
It was too expensive	2
Options were too overwhelming	3
Did not like available options	4
Decided to wait and reevaluate the situation later	5

Confidential. Do not distribute without permission.

Concerned about passing on a disease	6
Concerned about the baby's health	7
Other	8
[DO NOT READ]	
Don't know	99

E15. IF E9=1, YES AND IF E13=2 YES; Thinking about health information you or your family may have wanted about your fertility and your fertility preservation options, please say whether you strongly agree, agree, neither agree nor disagree, disagree, or strongly disagree with the following statement: I felt that my fertility preservation options were well explained:

Strongly agree	1
Agree	2
Neither agree nor disagree	3
Disagree	4
Strongly disagree	5

Medical Care for Infertility

Now I would like to talk to you about medical care for help getting pregnant. This could include tests to figure out if you can get a woman pregnant, treatments to help you get a woman pregnant, or procedures to help preserve your fertility.

E16. Have you ever visited a doctor or health professional for help getting your partner pregnant? Do not include times you talked to a doctor about fertility preservation for getting children in the future.

No	0	SKIP TO E40
Yes	1	

E17. IF E16=1, YES: The first time you went to see a doctor for help getting your partner pregnant, what was your primary reason for seeking help? (NOTE TO INTERVIEWER: IF MAN HAS BEEN TO SEE A DOCTOR AT MULTIPLE TIME POINTS FOR HELP GETTING HIS PARTNER PREGNANT, ASK THEM ABOUT THE FIRST TIME THEY WENT TO SEE THEIR DOCTOR. DON'T READ ANSWER CHOICES, BUT CHECK WHICH ANSWER CHOICES YOU FEEL CORRESPOND AND THEN READ BACK WHAT YOU CHECKED TO CONFIRM.)

Regular sex >1yr and not able to get your partner pregnant	1
Prior surgical or trauma history that made conceiving difficult	2
Prior history of chemotherapy or radiation treatment that may have caused infertility	3
Facing chemotherapy, radiation, or surgery that could impact my fertility	4
Female partner tied their tubes	5
I had a vasectomy	6
Had never tried to get a child but wanted to know if I could get a woman pregnant	7
Other	8

Confidential. Do not distribute without permission.

E18. IF E16=1, YES: What types of doctors or health professionals did you visit for help getting your partner pregnant? Please tell me all that apply. [NOTE TO INTERVIEWER – READ ALL OPTIONS]

A general practitioner	1	SKIP TO E20
A urologist	2	SKIP TO E20
A fertility specialist, such as a reproductive endocrinologist	3	GO TO E19
Another type of doctor	4	SKIP TO E20

E19. IF E16=1, YES: IF E18=3, A FERTILITY SPECIALIST, SUCH AS A REPRODUCTIVE ENDOCRINOLOGIST, READ: Were you <u>referred</u> to the fertility specialist by another doctor or health professional? [NOTE TO INTERVIEWER – IF SELF-REFERRED, MARK NO]

No	0	SKIP TO E22
Yes	1	SKIP TO E22

E20. IF E16=1, YES: IF [ANS E18]NE 3, READ: Were you referred to a fertility specialist by another doctor or health professional?

No	0	SKIP TO E22
Yes	1	

E21. IF E16=1, YES: IF [ANS E18]NE 3 AND [ANS E20]=1YES, READ: Why didn't you visit the fertility specialist initially ? Please tell me all that apply.

It was too expensive	1
It was too inconvenient	2
I did not know any fertility specialist	3
I did not know I needed to see a fertility specialist	4
I decided I only wanted to get a child naturally	5
I decided to adopt	6
I decided to stop trying to father a child	7
My partner did not want me to see a fertility specialist	8
Other	9

E22. IF D16=1 YES: At what age or ages did you visit a doctor or fertility specialist to help with getting your partner pregnant?
[NOTE TO INTERVIEWER, PROMPT FOR MONTHS AND YEARS IF CANNOT REMEMBER AGES. IF AN AGE RANGE IS GIVEN, ASK FOR BEST GUESS AS TO THE *FIRST* TIME SHE VISITED THE DOCTOR. IF VISITED THE DOCTOR OVER A WIDE RANGE OF AGES (>2 YEARS), MARK THE YOUNGEST AGE AND ASK IF THERE WERE ANY OTHER TIMES WHEN THEY STARTED A NEW FERTILITY VISITATION CYCLE; RECORD ANY OTHER AGES]

Age1: _____
Age2: _____

Date1: ________ (MM/ YYYY)

Confidential. Do not distribute without permission.

Date2: ________ (MM/ YYYY)

Fertility Tests

E23. IF E16=1, YES: After talking to your doctor or fertility specialist, did you ever have tests to check your ability to get a woman pregnant?

No	0	SKIP TO E25
Yes	1	

E24. IF E23=1, YES: Did you have any of the following types of fertility tests done? Please answer yes or no:

[NOTE TO INTERVIEWER – PLEASE READ ANSWER CHOICE AND WAIT FOR A RESPONSE FOR EACH OPTION; READ THROUGH ENTIRE LIST.]

Tests to check your semen and determine sperm count and other sperm characteristics

No 0
Yes 1
[DO NOT READ]
Don't know 99

IF YES, THEN READ: How old were you when you had these tests done? If this occurred more than once, please tell me all of the ages when you had these tests done. [NOTE TO INTERVIEWER, IF PROVIDES AGE RANGE, PROMPT FOR BEST GUESS. PROMPT FOR MONTHS AND YEARS IF CANNOT REMEMBER AGES]

Age1: _____
Age2: _____

Blood test for reproductive hormones to investigate if you had any issues with sperm production

No 0
Yes 1

[DO NOT READ]
Don't know 99

IF YES, THEN READ: How old were you when you had these blood tests? [NOTE TO INTERVIEWER, PROMPT FOR MONTH AND YEAR IF CANNOT REMEMBER AGE]

Age(s): ____
Month(s): ____
Year(s): ____

Confidential. Do not distribute without permission.

Any other tests

No	0
Yes	1

[DO NOT READ]
Don't know 99

IF YES, THEN READ: How old were you when you had these other tests? [NOTE TO INTERVIEWER, PROMPT FOR MONTH AND YEAR IF CANNOT REMEMBER AGE]

Age(s): ____
Month(s): ____
Year(s): ____

E25. Were you ever told by a medical professional that there was a problem with your fertility or your partner's fertility that would affect your ability to father a child? [NOTE TO INTERVIEWER – IF COUPLE HAS UNEXPLAINED INFERTILITY, CHOOSE ANSWER OPTION 2]

No	0	SKIP TO E28
Yes	1	
The exact reason was never determined	2	SKIP TO E28

E26. IF E25=1, YES: What were you told? Please tell me all that apply.

Female partner had problems, such as blocked tubes, ovulation problem or uterus problem	1
Problem with sperm such as low count, low motility or other sperm problem	2
Another reason	3

E27. IF E25=1, FEMALE PARTNER HAD PROBLEM, READ: How old were **YOU** when your partner was told that there was a problem with her fertility? If this happened more than once, please tell me all of the ages when you were told this.
[NOTE TO INTERVIEWER, IF PROVIDES AGE RANGE, PROMPT FOR BEST GUESS. PROMPT FOR MONTHS AND YEARS IF CANNOT REMEMBER AGES]

Age1: _____
Age2: _____

Date1: ________ (MM/ YYYY)
Date2: ________ (MM/ YYYY)

Infertility Treatments

Confidential. Do not distribute without permission.

E28. IF E16=1 YES: Did you *ever* pursue treatment to help you get your partner pregnant? This includes any medications to help you improve your sperm count, or other treatments such surgery, as intrauterine insemination or in vitro fertilization. Only include surgeries that were done specifically to help you get a woman pregnant.
During **IVF**, mature eggs are collected (retrieved) from ovaries and fertilized by sperm in a lab. Then the fertilized egg (embryo) or eggs (embryos) are transferred to your partners uterus. **IUI** stands for in **intrauterine insemination**. It's also sometimes called donor insemination, alternative insemination, or artificial insemination. **IUI works** by putting sperm cells directly into your partner's uterus around the time she is ovulating, helping the sperm get closer to the egg.

No	0	
Yes	1	SKIP TO E30

E29. IF E16=1, YES AND IF [ANS E28]=0: What are some reasons you decided not to pursue treatment to help you get your partner pregnant? Please tell me all that apply.

It was too expensive	1
Did not like the treatment options	2
Partner did not want to participate in treatment	3
Got my partner pregnant without treatment	4
Decided to try to father a child naturally without treatment	5
Decided to adopt children	6
Decided it was not the right time in your life to father children	7
Decided not to have children	8
Doctor didn't know what the exact problem was	9
Another reason	10

IF E28**=0, NO, SKIP TO** E40

Surgery

E30. IF E28=1, YES, READ: Did you ever have surgery to help you get a woman pregnant? This could include surgery to fix blocked sperm ducts and or testicular varicocele veins. Do not include surgical sperm retrieval in this answer.

No	0	SKIP TO E32
Yes	1	

E31. At what age did you first have surgery to help you get a woman pregnant?
[NOTE TO INTERVIEWER, IF PROVIDES AGE RANGE, PROMPT FOR BEST GUESS. PROMPT FOR MONTH AND YEAR IF CANNOT REMEMBER AGE]

Age: _____
Month: _____
Year: _____

Confidential. Do not distribute without permission.

Medications

E32. IF E28=1, YES. Did you ever take any medication or hormones, including shots, to help you get a woman pregnant and or improve your sperm count? This could include oral medications or injectable medications. E36

No	0	SKIP TO E35
Yes	1	

E33. At what age did you first take *these* medications to help you get a woman pregnant and or improve your sperm count?
[NOTE TO INTERVIEWER, IF PROVIDES AGE RANGE, PROMPT FOR BEST GUESS. PROMPT FOR MONTH AND YEAR IF CANNOT REMEMBER AGE]

Age: ____
Month: ____
Year: ____

E34. IF E28=1, YES At this time, did you get your partner pregnant? Include pregnancies ending in miscarriage or still birth.

No	0	SKIP TO E35
Yes	1	

Insemination

E35. IF E28=1, YES Did you ever have artificial or intrauterine insemination? This involves having sperm injected into your partner's uterus to help her get pregnant.

No	0	SKIP TO E40
Yes	1	

E36. IF E28=1, YES, IF E35.=1, YES. At what age was this first done?
[NOTE TO INTERVIEWER, IF PROVIDES AGE RANGE, PROMPT FOR BEST GUESS. PROMPT FOR MONTH AND YEAR IF CANNOT REMEMBER AGE.]

Age: ____
Month: ____
Year: ____

E37. Did your partner get pregnant by using artificial insemination at this time; this would include a pregnancy ending in miscarriage or still birth? [NOTE TO INTERVIEWER – IF RESPONDS THAT SHE HAD A STILLBIRTH, NOTE THAT UNDER 'YES']

No	0	SKIP TO E40
Yes	1	

Confidential. Do not distribute without permission.

E38. Did you ever have artificial or intrauterine insemination again?

No	0	SKIP TO E40
Yes	1	

E39. At what age did you next have this done?
[NOTE TO INTERVIEWER, IF PROVIDES AGE RANGE, PROMPT FOR BEST GUESS. PROMPT FOR MONTH AND YEAR IF CANNOT REMEMBER AGE.]

Age: ____
Month: ____
Year: ____
RETURN TO E36

Sperm use – Introduction

E40. Have you ever tried to have your sperms used? This could be done for fertility procedures such as in vitro fertilization, for fertility preservation (sperm or embryo freezing), or to donate your sperm to another man. Please tell me all that apply.

No	0	SKIP TO E69
Yes, for in vitro fertilization or Intrauterine insemination	1	
Yes, for fertility preservation	2	
Yes, to donate sperms to another man	3	

IF E40 NE 0. I am going to ask you about each of your sperm use cycles separately, but first, I want you to think about ***all*** of the cycles where you tried to have your sperm used. This may include several cycles in a row or it may include cycles from different attempts to use your sperms. It also may include cycles where they were unable to use your sperms.

E41. Altogether, how many cycles have you tried to have your sperms used? [NOTE TO INTERVIEWER – THIS SHOULD BE THE TOTAL NUMBER OF CYCLES A MAN HAD ATTEMPTED TO HIS SPERM USED, WHETHER OR NOT IT WAS SUCCESSFUL.]

Number of cycles: ____

IF [ANS E40] NE 1 AND NE 0: THEN SKIP TO INSTRUCTIONS BEFORE E54

Sperm use– In Vitro Fertilization

E42. IF [ANS E40]=1: At, what age did you first try to have your sperm used for in vitro fertilization or intrauterine insemination?
[NOTE TO INTERVIEWER, IF PROVIDES AGE RANGE, PROMPT FOR BEST GUESS. PROMPT FOR MONTH AND YEAR IF CANNOT REMEMBER AGE.]

Age: ____

Confidential. Do not distribute without permission.

Month: ____
Year: ____

E43. At this time, were they able to use your sperm?

No	0	
Yes	1	SKIP TO E45

E44. IF E43=0, NO, READ: Why not?

Did not have any sperm in your semen	1	SKIP TO E69
Unable to produce sperm	2	SKIP TO E69
Other	3	SKIP TO E69
[DO NOT READ]		
Don't know	99	SKIP TO E69

E45. IF E43= 1, YES; Did you use eggs [for an IVF] from your partner, someone you knew, or an anonymous donor or surrogate [in case of for IUI]?

Partner	1
Donor you knew	2
Anonymous donor	3
Surrogate [IUI]	4

E46. IF E43= 1 AND E45 NE 4 Were the embryos transferred to your partner or to a surrogate?

You	0
A surrogate	1

NOTE TO INTERVIEWER – IF MAN SAYS CYCLE WAS CANCELLED PRIOR TO THE TRANSFER, SELECT 'NOT TRANSFERRED, 97' FOR E47 AND SKIP TO E49

E47. IF E43= 1, AND E45 NE 4 How many fresh embryos were transferred during this cycle? [NOTE TO INTERVIEWER – IF PROVIDES A RANGE OF EMBRYOS TRANSFERRED, ASK FOR BEST GUESS.]

Number: ____

[DO NOT READ]		
Don't know	99	
Not transferred	97	SKIP TO E49

E48. Did the treatment result in a pregnancy during this cycle; this would include a pregnancy ending in a miscarriage or still birth?

No	0	SKIP TO E49
Yes	1	

Confidential. Do not distribute without permission.

E49. IF E43= 1, AND E45 NE 4 Did you freeze embryos from this IVF cycle?

No	0	SKIP TO E51
Yes	1	
[DO NOT READ]		
Don't know	99	

E50. IF E43= 1, AND E45 NE 4 How many total embryos did you freeze? [NOTE TO INTERVIEWER – IF PROVIDES A RANGE OF EMBRYOS FROZEN, PROMPT FOR BEST GUESS.]

Number: ____

IF E49 = 0, NO, SKIP TO INSTRUCTIONS BEFORE E54

E51. IF E43= 1, AND E45 NE 4 AND IF E49 =1, YES, READ: Did you ever have any of your frozen embryos transferred to your partner or a surrogate?

No	0	SKIP TO INSTRUCTIONS BEFORE E54
Yes, to my partner	1	
Yes, to a surrogate	2	

E52. IF E49=1, YES, TO MY PARTNER OR E49=2, YES, TO A SURROGATE READ: How many frozen embryos were transferred at that time? [NOTE TO INTERVIEWER, IF PROVIDES RANGE OF FROZEN EMBRYOS, PROMPT FOR BEST GUESS. IF MAN SAYS CYCLE WAS CANCELLED PRIOR TO THE TRANSFER, SELECT 'NOT TRANSFERRED, 97' AND SKIP TO F61.]

Number: ____

[DO NOT READ]		
Don't know	99	
Not transferred	97	SKIP TO INSTRUCTIONS BEFORE E54

E53. IF E49=1, YES, TO MY PARTNER OR E49=2, YES, TO A SURROGATE READ Did the transfer result in a pregnancy; this would include a pregnancy ending in miscarriage or still birth?

No	0	SKIP TO INSTRUCTIONS BEFORE E54
Yes	1	

Confidential. Do not distribute without permission.

IF [ANS E40] NE 2 THEN SKIP TO INSTRUCTIONS BEFORE E69

E54. IF [ANS E40]=2: At what age did you first ***try*** to have your sperms used for fertility preservation? This could be at the time of a cancer diagnosis or at another time. [NOTE TO INTERVIEWER, IF PROVIDES AGE RANGE, PROMPT FOR BEST GUESS. PROMPT FOR MONTH AND YEAR IF CANNOT REMEMBER AGE.]

Age: ____
Month: ____
Year: ____

E55. At this time, were they able to use your sperms?

No	0	
Yes	1	SKIP TO E57

E56. IF E55=0, NO, READ: Why not?

Did not have any sperm in your semen	1	
Unable to produce sperm	2	
Other	3	
[DO NOT READ]		
Don't know	99	

E57. IF E55=1 YES, when you had this sperm used for fertility preservation done, did you freeze sperms or embryos (these are fertilized eggs), or both sperms and embryos? [NOTE TO INTERVIEWER, IF HE HAD BOTH SPERMS AND EMBRYOS FROZEN, CHOOSE OPTIONS 1 AND 2. IF HE KNOWS THAT HE HAD EMBRYOS FROZEN BUT IS UNSURE IF HE HAD SPERM FROZEN CHOOSE OPTION 2.]

Sperm	1	
Embryos	2	
Sperm and Embryos	3	
[DO NOT READ]		
Don't know	99	

IF [ANS E57 NE 1, SPERM] SKIP TO INSTRUCTIONS BEFORE E63

E58. IF **E57**=1, SPERM, READ: Did you later thaw the sperm to fertilize eggs and use fertilized eggs for an embryo transfer to you partner or a surrogate?

No	0	SKIP TO E63
Yes, to your partner	1	
Yes, to a surrogate	2	

Confidential. Do not distribute without permission.

IF **E57**=1, SPERM, READ: Did you later thaw the sperm to use in an intrauterine insemination treatment of your partner or surrogate?

No	0	SKIP TO E63
Yes, to your partner	1	
Yes, to a surrogate	2	

E59. IF E58=1, YES TO YOUR PARTNER OR E58=2, YES TO A SURROGATE READ: How old were **you** at that time? [NOTE: IF THE MAN SAYS HE USED THE SPERM MORE THAN ONCE, ASK HIM ABOUT HIS AGE THE FIRST TIME.]
[NOTE TO INTERVIEWER, IF PROVIDES AGE RANGE, PROMPT FOR BEST GUESS. PROMPT FOR MONTH AND YEAR IF CANNOT REMEMBER AGE]

Age: _____
Month: _____
Year: _____

E60. IF E58=1, YES TO ME OR E58=2, YES TO A SURROGATE BUT NOT IUI READ Did you use eggs from your partner, someone you knew, or an anonymous donor?

Partner	1
Donor you knew	2
Anonymous donor	3

E61. IF E58=1, YES TO ME OR E58=2, YES TO A SURROGATE BUT NOT READ How many embryos were transferred? [NOTE TO INTERVIEWER – IF MAN PROVIDES A RANGE OF EMBRYOS TRANSFERRED, PROMPT FOR BEST GUESS. IF MAN SAYS CYCLE WAS CANCELLED PRIOR TO THE TRANSFER, SELECT 'NOT TRANSFERRED, 97]

Number: _____

[DO NOT READ]

Don't know	99
Not transferred	97

E62. IF E58=1, YES TO ME OR E58=2, YES TO A SURROGATE READ Did this treatment result in a pregnancy; this would include a pregnancy ending in miscarriage or still birth?

No	0
Yes	1

IF [ANS E57 NE 2] SKIP TO INSTRUCTIONS BEFORE E69

E63. IF **E57** =2, EMBRYOS, READ: How many embryos did you have frozen? [NOTE TO INTERVIEWER – IF MAN PROVIDES A RANGE OF EMBRYOS FROZEN, PROMPT FOR BEST GUESS.]

Confidential. Do not distribute without permission.

Number: ____

E64. IF **E57** =2, EMBRYOS, READ: Did you use eggs from your partner, someone you knew, or an anonymous donor?

Partner	1
Donor you knew	2
Anonymous donor	3

E65. IF **E57** =2, EMBRYOS, READ: Did you later have an embryo transfer to your partner or a surrogate?

No	0
Yes, to my partner	1
Yes, to a surrogate	2

E66. IF E65=1, YES TO MY PARTNER OR E65=2, YES TO A SURROGATE, READ: How old were **you** at the time? [NOTE: IF THE MAN SAYS SHE USED THE EMBRYOS MORE THAN ONCE, ASK HER ABOUT HER AGE THE FIRST TIME.]
[NOTE TO INTERVIEWER, IF PROVIDES AGE RANGE, PROMPT FOR BEST GUESS. PROMPT FOR MONTH AND YEAR IF CANNOT REMEMBER AGE.]

Age: ____
Month: ____
Year: ____

E67. IF E65=1, YES TO MY PARTNER OR E65=2, YES TO A SURROGATE :How many embryos were transferred? [NOTE TO INTERVIEWER – IF PROVIDES A RANGE OF EMBRYOS, PROMPT FOR BEST GUESS. IF MAN SAYS CYCLE WAS CANCELLED PRIOR TO THE TRANSFER, SELECT 'NOT TRANSFERRED, 97' AND SKIP TO E69

Number: ____
[DO NOT READ]

Don't know	99
Not transferred	97

E68. IF E65=1, YES TO MY PARTNER OR E65=2, YES TO A SURROGATE Did this transfer result in a pregnancy; this would include a pregnancy ending in a miscarriage or a still birth?

No	0
Yes	1

Confidential. Do not distribute without permission.

Donor sperms

E69. **IF [ANS E40] NE 3** Have you ever used donor sperm for in vitro fertilization or intrauterine insemination to help you partner or a surrogate get pregnant?

No	0	SKIP TO INSTRUCTIONS BEFORE F1
IVF	1	
IUI	2	

E70. IF E69 NE 0, How old were **you** the first time?
[NOTE TO INTERVIEWER, IF PROVIDES AGE RANGE, PROMPT FOR BEST GUESS. PROMPT FOR MONTH AND YEAR IF CANNOT REMEMBER AGE.]

Age: ____
Month: ____
Year: _____

E71. IF E69 NE 0Was the donation by a friend, a relative, or an anonymous donor?

Friend	1
Relative	2
Anonymous donor	3
Other	4

E72. IF E69 NE 0. For In-vitro fertilization [E69 = 1]: Did you use eggs from your partner, someone you knew, or an anonymous donor?

Partner	1
Donor you knew	2
Anonymous donor	3

For intrauterine insemination [E69 = 2]: Did use your partner's uterus or that of a surrogate you knew or an anonymous surrogate

Partner	1
Surrogate you knew	2
Anonymous surrogate	3

E73. IF E69=1 Were the embryos transferred to your partner or a surrogate?

Your partner	1
A surrogate	2

Confidential. Do not distribute without permission.

E74. IF E69 =1 How many embryos were transferred? [NOTE TO INTERVIEWER, IF PROVIDES A RANGE OF EMBRYOS TRANSFERRED, PROMPT FOR BEST GUESS. IF MAN SAYS CYCLE WAS CANCELLED PRIOR TO THE TRANSFER, SELECT 'NOT TRANSFERRED, 97' AND SKIP TO INSTRUCTIONS BEFORE F1]

Number: ____

[DO NOT READ]

Don't know	99	
Not transferred	97	SKIP TO INSTRUCTIONS BEFORE F1

E75. IF E69 NE 0, Did this treatment result in a pregnancy; this would include a pregnancy ending in a miscarriage or still birth?

No	0	SKIP TO INSTRUCTIONS BEFORE F1
Yes	1	

SECTION F: PREGNANCY HISTORY

Now, I would like to ask you some questions about any pregnancies you have fathered.

F1. How many times have you been responsible for a pregnancy including miscarriages, still births, tubal pregnancies, abortions, and live births?

Number of pregnancies ____

IF ANS F1=0 SKIP TO INSTRUCTIONS BEFORE G1

F2. How old were you when you got a woman pregnant the first time?
[NOTE TO INTERVIEWER, IF PROVIDES AGE RANGE, PROMPT FOR BEST GUESS. PROMPT FOR MONTH AND YEAR IF CANNOT REMEMBER AGE.]

Age: ____
Month: ____
Year: ____

IF ANS E28=0, NO, SKIP TO F7

F3. IF **E28** NE 0, READ: Did you undergo some form of medical treatment to help you get your partner pregnant this first time?

No	0	SKIP TO F7
Yes	1	

Confidential. Do not distribute without permission.

F4. IF ANS F3= 1, YES, READ: What treatment came just before you got your partner pregnant?

Intrauterine insemination	1
Fertility medications with intercourse	2
Assisted Reproductive Technologies	3
Other	4

F5. For this pregnancy, did you try to get your partner pregnant naturally before using fertility treatments?

No	0	SKIP TO F7
Yes	1	

F6. IF ANS F5=1, YES, READ: How long did you try to get your partner pregnant before seeking fertility treatment? [NOTE TO INTERVIEWER – IF PROVIDES A RANGE OF MONTHS OR YEARS, PROMPT FOR BEST GUESS.]

Months: ____
Years: ____

SKIP TO INSTRUCTIONS BEFORE G1

F7. IF ANS **E28** =0, NO OR IF ANS F3=0, NO, READ: Were you and or your partner using any form of birth control when you got her pregnant *this* time?

No	0	
Yes	1	SKIP TO INSTRUCTIONS BEFORE G1

F8. Were you trying to get your partner pregnant?

No	0
Neither trying nor not trying	1
Yes	2

F9. How long were you having regular unprotected sex before you got your partner pregnant? Regular intercourse means at least 3 times per month. [NOTE TO INTERVIEWER – IF PROVIDES A RANGE OF MONTHS AND YEARS, PROMPT FOR BEST GUESS. IF STILL CANNOT GIVE SINGLE ANSWER, READ OPTIONS.]

Months: ____
Years: ____

[PROMPT IF RESPONDENT DOES NOT KNOW]: Was it . . .

1-3 Months	1
4-6 Months	2

Confidential. Do not distribute without permission.

7-9 Months	3
10-12 Months	4
More than a year	5

SECTION G: LIFESTYLE

Now, I want to ask you some questions about your lifestyle. This is the last section of the interview.

Smoking History

ACTIVE SMOKING

G1. Have you ever smoked at least 100 cigarettes in your lifetime?

No	0
Yes	1

G2. Have you ever smoked or used any of the following at least 20 times in your lifetime? Please say yes or no to each. [NOTE TO INTERVIEWER – IF ASKED, PIPES AND WATER PIPES/SHISHA ARE NOT THE SAME.]

Pipes	1
Chewing tobacco	2
Marijuana	3
Shisha water pipe	4
None of these	0

IF G1=0, NO SKIP TO INSTRUCTIONS BEFORE G8

G3. IF **G1**=1, YES: Have you ever smoked cigarettes on a regular basis? That is, have you ever smoked an *average* of 7 or more cigarettes a week for six months or longer?

No	0	SKIP TO INSTRUCTIONS BEFORE G8
Yes	1	
[DO NOT READ] Don't know	99	SKIP TO INSTRUCTIONS BEFORE G8

G4. IF G3=1, YES, READ: At what age did you first start smoking cigarettes on a regular basis? [NOTE TO INTERVIEWER, IF PROVIDES AGE RANGE, PROMPT FOR BEST GUESS. PROMPT FOR MONTH AND YEAR IF CANNOT REMEMBER AGE.]

Age: ____

Confidential. Do not distribute without permission.

Month: ____
Year: ____

G5. Throughout your lifetime, approximately how many years did you smoke cigarettes regularly? Do not count periods of time when you may have quit. [NOTE TO INTERVIEWER – IF PROVIDES A RANGE OF YEARS, PROMPT FOR BEST GUESS.]

Years: ____

[DO NOT READ]
Less than one year 97

G6. During the times you regularly smoked cigarettes, how many cigarettes did you smoke on average per week? [NOTE TO INTERVIEWER – IF PROVIDES A RANGE OR CIGARETTES, PROMPT FOR BEST GUESS. IF ANSWERS IN NUMBER OF PACKS PER WEEK, PLEASE CONVERT TO NUMBER OF CIGARETTES PER WEEK USING THE FORMULA:

[NUMBER OF CIGARETTES = NUMBER OF PACKS * 20]

Number: _____

G7. Do you currently smoke regularly?

No 0
Yes 1

IF G2 NE 1, SKIP TO INSTRUCTIONS BEFORE G13

G8. IF G2=1, PIPES: Have you ever smoked a pipe on a regular basis? That is, have you ever smoked an average of at least one pipe each week for a year or more?

No 0 SKIP TO INSTRUCTIONS BEFORE G13
Yes 1

[DO NOT READ]
Don’t know 99 SKIP TO INSTRUCTIONS BEFORE G13

G9. IF G2=1, YES, READ: At what age did you start smoking a pipe on a regular basis? [NOTE TO INTERVIEWER, IF PROVIDES AGE RANGE, PROMPT FOR BEST GUESS. PROMPT FOR MONTH AND YEAR IF CANNOT REMEMBER AGE.]

Age: ____
Month: ____
Year: ____

Confidential. Do not distribute without permission.

G10. Throughout your lifetime, approximately how many years did you regularly smoke a pipe? Do not count periods of time when you may have quit. [NOTE TO INTERVIEWER – IF PROVIDES A RANGE OF YEARS, PROMPT FOR BEST GUESS.]

Years: ____

[DO NOT READ]
Less than one year 97

G11. During the times you regularly smoked pipes, how many pipes did you smoke on average each week? [NOTE TO INTERVIEWER – IF PROVIDES A RANGE OF PIPES, PROMPT FOR BEST GUESS.]

Number: _____

G12. Do you currently smoke pipes regularly?

No 0
Yes 1

IF G2 NE 2, SKIP TO INSTRUCTIONS BEFORE G18

G13. IF G2=2, CHEWING TOBACCO: Have you ever chewed tobacco on a regular basis? That is, have you ever chewed tobacco at least once a week for a year or more?

No 0 SKIP TO INSTRUCTIONS BEFORE G18
Yes 1

[DO NOT READ]
Don't know 99 SKIP TO INSTRUCTIONS BEFORE G18

G14. IF G13=1, YES, READ: At what age did you start chewing tobacco on a regular basis? [NOTE TO INTERVIEWER, IF PROVIDES AGE RANGE, PROMPT FOR BEST GUESS. PROMPT FOR MONTH AND YEAR IF CANNOT REMEMBER AGE.]

Age: ____
Month: ____
Year: ____

Confidential. Do not distribute without permission.

G15. Throughout your lifetime, approximately how many years did you chew tobacco regularly? Do not count periods of time when you may have quit. [NOTE TO INTERVIEWER – IF PROVIDES A RANGE OF YEARS, PROMPT FOR BEST GUESS.]

Years: ____

[DO NOT READ]
Less than one year 97

G16. During the times you have regularly chewed tobacco, how many tins did you chew on average each week? [NOTE TO INTERVIEWER – IF PROVIDES A RANGE OF TINS, PROMPT FOR BEST GUESS.]

Number: _____

G17. Do you currently chew tobacco regularly?

No 0
Yes 1

IF G2 NE 3, SKIP TO INSTRUCTIONS BEFORE G23

G18. IF G2=3, MARIJUANA OR POT: Have you ever used marijuana on a regular basis? That is on average at least once a week for a year or more?

No 0 SKIP TO **G23**
Yes 1

[DO NOT READ]
Don't know 99 SKIP TO **G23**

G19. IF G18=1, YES, READ: At what age did you start using marijuana on a regular basis? [NOTE TO INTERVIEWER, IF PROVIDES AGE RANGE, PROMPT FOR BEST GUESS. PROMPT FOR MONTH AND YEAR IF CANNOT REMEMBER AGE.]

Age: ____
Month: ____
Year: ____

G20. Throughout your lifetime, approximately how many years did you use marijuana on a regular basis? Do not count periods of time when you may have stopped using marijuana. [NOTE TO INTERVIEWER – IF PROVIDES A RANGE OF YEARS, PROMPT FOR BEST GUESS.]

Years: ____
[DO NOT READ] Less than one year 97

G21. When you were regularly using marijuana, on average, how many times per week did you use marijuana? [IF PROVIDES A RANGE OF TIMES, PROMPT FOR BEST GUESS.]

Number of times: ____

Confidential. Do not distribute without permission.

G22. Do you currently use marijuana?

No 0
Yes 1

IF G2 NE 4, SKIP TO G28

G23. IF G2=5, SHISHA OR WATER PIPES: Have you ever used Shisha or water pipes on a regular basis? That is, have you ever used shisha or water pipe on average once a week for a year or more?

No 0 SKIP TO INSTRUCTIONS BEFORE **G28**
Yes 1

[DO NOT READ]
Don't know 99 SKIP TO INSTRUCTIONS BEFORE **G28**

G24. IF G23=1, YES, READ: At what age did you start using shisha or water pipe on a regular basis?
[NOTE TO INTERVIEWER, IF PROVIDES AGE RANGE, PROMPT FOR BEST GUESS. PROMPT FOR MONTH AND YEAR IF CANNOT REMEMBER AGE.]

Age: ____
Month: ____
Year: ____

G25. Throughout your lifetime, approximately how many years did you regularly use shisha or water pipe? Do not count periods of time when you may have stopped using shisha or water pipe. [NOTE TO INTERVIEWER – IF PROVIDES A RANGE OF YEARS, PROMPT FOR BEST GUESS.]

Years: ____

[DO NOT READ]
Less than one year 97

G26. During the times you regularly used shisha or water pipe, on average, how many times per week did you use shisha or water pipe? [NOTE TO INTERVIEWER – IF PROVIDES A RANGE OF USE, PROMPT FOR BEST GUESS.]

Number: _____

Confidential. Do not distribute without permission.

G27. Do you currently smoke pipes regularly?

No	0	
Yes	1	

Alcohol Consumption

Now I will ask you some questions about your alcohol consumption. Please answer about your alcohol consumption.

G28. Have you ever consumed at least 20 alcoholic beverages in your lifetime?

No	0	SKIP TO INSTRUCTIONS BEFORE G32
Yes	1	

G29. Currently, how often do you drink an alcoholic beverage, by that I mean at least one beer, one glass of wine, one mixed drink, or one shot of liquor? You may answer in number of times per day, week, month, or year. [NOTE TO INTERVIEWER – IF PROVIDES A RANGE OF OCCAISIONS, PROMPT FOR BEST GUESS.]

Never	0	SKIP TO G32
Everyday	1	
Times/week	2	
Times/month	3	
Times/year	4	

If 2, 3, or 4 then Number of times: _______

G30. Currently, on those occasions that you drink alcoholic beverages, how many drinks do you usually have in one sitting? [NOTE TO INTERVIEWER - IF PROVIDES A RANGE OF DRINKS, PROMPT FOR BEST GUESS.]

Drinks/occasion: ______

G31. How old were you when you started drinking alcohol? [NOTE TO INTERVIEWER, IF PROVIDES AGE RANGE, PROMPT FOR BEST GUESS. PROMPT FOR MONTH AND YEAR IF CANNOT REMEMBER AGE.]

Age: _____
Month: _____
Year: _____

Income

Now I am going to ask you some questions about your job, and your income.

Confidential. Do not distribute without permission.

G32. Are you employed? Include part-time and full-time jobs

No	0
Yes, full time	1
Yes, part time	2

G33. Which of the following best describes your occupation?

Drop down list: 1-10

G34. In the past 12 months, what was your total household income? Please include sum for all sources of income.

TEXT BOX: _______

G35. How many people are supported by this income, including adults and children?

Number of people: _____

SECTION H: CONCLUSION & CONTACT INFORMATION

Thank you for participating in the URHSPY Cancer Research study. We will be sending you a $3 airtime gift card to thank you for your time. Before we send the gift card, we need to confirm that we are spelling your name correctly and that we have your current contact information. This information will be stored in a separate password protected file from your answers to the interview questions.

H1. Your name and address are: [INTERVIEWER: IF COMPLICATED SPELLING OF ANY, PLEASE SPELL IT BACK TO THEM]

First Name Last Name
Address

H2. Do you have an email address?

Email address

H3. The phone numbers I have are:

Cell 1
Cell 2

Confidential. Do not distribute without permission.

Is this correct?

No	0	
Yes	1	SKIP TO H7

H4. IF H3 =0, NO READ: Which numbers were incorrect?

Cell 1	1
Cell 2	2

H5. IF H4=1, CELL 1, READ: What is the correct number for your cell phone?

Cell 1 phone number:

H6. IF H4=2, CELL 2, READ: What is the correct number for your second cell phone?

Cell 2 phone number:

H7. Is there another number where we can reach you?

No	0
Yes	1

H8. IF H7=1, YES, READ: What is that number?

Phone number:

H9. Is that a home, work, or cellphone number?

Home	1
Work	2
Cell	3

SECTION I: INTERVIEWER IMPRESSION

I1. End of interview

Time

I2. Duration of interview

Minutes [BEFORE A1– ANS I1]

I3. Number of sessions needed to complete interview

1

Confidential. Do not distribute without permission.

2
3 or more

I4. The overall quality of this interview was

High: Participant was absolutely certain about all the information, was clear about all the various behaviors, dates, and so forth	1	SKIP TO I6
Generally reliable: Participant was engaged, responded to all questions but hesitated on some answers. Needed to think through dates, etc...	2	SKIP TO I6
Questionable: Participant didn't understand some of the questions, was not paying attention to part of the interview, or was distracted.	3	
Unsatisfactory: Participant appeared to be making up most of the answers, or she couldn't understand most of the questions, or was not paying attention to most of the interview.	4	

I5. The main reason the interview was of questionable or unsatisfactory quality was because

Didn't know enough info regarding the topic	1
Did not want to be specific	2
Sounded bored or uninterested	3
Sounded upset, depressed, angry	4
Had poor hearing or speech	5
Sounded distracted or confused	6
Frequent interruptions	7
Sounded embarrassed by the subject	8
Sounded emotionally unstable	9
Sounded physically ill	10
Not comfortable with the language of the questionnaire	11
Didn't have time	12
Felt interview was too long	13
Other: ___________________________	14

I6. Other comments that may affect the interpretation of the respondent's answers?

Comments

Confidential. Do not distribute without permission.
