# Supplementary material for: Understanding Reproductive Health among Survivors of Paediatric and Young adults (URHSPY) cancers in Uganda: A mixed method study protocol
Source: PLoS One. 2023 Apr 25;18(4):e0284969. doi: 10.1371/journal.pone.0284969 (PMC10128918; doi:10.1371/journal.pone.0284969)
Supplement: Attachment — Submitted filename: Response to reviewers .docx [file pone.0284969.s004.docx]

Wednesday, November 2, 2022

**Editor-in-Chief**

**PLOS ONE journal**

Dear sir or madam,

**RE: Response to reviewers for protocol # PONE-D-22-20391; Understanding Reproductive Health among Survivors of Pediatric and Young adults (URHSPY) Cancers in Uganda: a mixed method study protocol**
Thank you for accepting to review our manuscript. We have prepared a point-by-point response to the reviewers concerns in the body on this letter. Changes in the manuscript are tracked, and where needed highlighted in red.

Journal requirements

1. **Comment:** When submitting your revision, we need you to address these additional requirements. Please ensure that your manuscript meets PLOS ONE's style requirements, including those for file naming. The PLOS ONE style templates can be found at
   <https://journals.plos.org/plosone/s/file?id=wjVg/PLOSOne_formatting_sample_main_body.pdf> and <https://journals.plos.org/plosone/s/file?id=ba62/PLOSOne_formatting_sample_title_authors_affiliations.pdf>

**Response:** The manuscript style and file naming has been modified to meet PLOS ONE's requirements.

2. **Comment:** Thank you for stating the following financial disclosure: "This study is funded by the North Pacific Global Health (NPGH) research fellow training consortium award to A.K, under the Fogarty International Centre and the National Institutes of Health [Grant # D43 TW009345] (<https://www.fic.nih.gov/>) and supported by the National Institutes of Health's National Centre for Advancing Translational Sciences [Grant # UL1 TR002494] ([https://www.nih.gov/about-nih/what-we-do/nih-almanac/national-center-advancing-translational-sciences. ncats#:~:text=NCATS'%20mission%20is%20to%20catalyze,improving%20health%20through%20smart%20science.](https://www.nih.gov/about-nih/what-we-do/nih-almanac/national-center-advancing-translational-sciences.%20ncats#:~:text=NCATS'%20mission%20is%20to%20catalyze,improving%20health%20through%20smart%20science.)). The funding organizations did not have any role in the design of the study or writing the manuscript. They will not be involved in data collection, analysis, and interpretation of data." Please state what role the funders took in the study.  If the funders had no role, please state: "The funders had no role in study design, data collection and analysis, decision to publish, or preparation of the manuscript." If this statement is not correct you must amend it as needed.
   Please include this amended Role of Funder statement in your cover letter; we will change the online submission form on your behalf.

**Response:** We have amended the financial disclosure statement to include: “The funders had no role in study design, data collection and analysis, decision to publish, or preparation of the manuscript.” [see page 14, under funding section].

3. **Comment:** Your ethics statement should only appear in the Methods section of your manuscript. If your ethics statement is written in any section besides the Methods, please move it to the Methods section and delete it from any other section. Please ensure that your ethics statement is included in your manuscript, as the ethics statement entered into the online submission form will not be published alongside your manuscript.

**Response:** The ethics section has been moved it to the Methods section and deleted from any other section. [see page 6, first section/paragraph].

**REVIEWER 1**

1. **Comment:** Is the manuscript presented in an intelligible fashion and written in standard English? PLOS ONE does not copyedit accepted manuscripts, so the language in submitted articles must be clear, correct, and unambiguous. Any typographical or grammatical errors should be corrected at revision, so please note any specific errors here. Reviewer #1: No.

**Response:** Extensive efforts have been made to edit grammar and spelling throughout the manuscript. The paragraphs have also been reordered and revised to succinctly convey the message of the manuscript.

2. **Comment:** Overall, the protocol manuscript would significantly benefit from editorial assistance for grammar and punctuation corrections in each paragraph. It is also repetitive in some sections.

**Response:** Extensive efforts have been made to edit grammar and spelling throughout the manuscript. The paragraphs have also been reordered and revised to succinctly convey the message of the manuscript.

3. **Comment:** Abstract Methods: Please include the estimated sample size for both the survey and interview components.

**Response:** The sample size for both study designs have been included in the manuscript abstract. [see page 2, under methods, lines 5 and 8]

4. **Comment:** Background: This section would benefit from a re-organization to concisely present the rationale for the study’s purpose. The first paragraph can be removed as it is not necessary to present the global cancer incidence. Suggestion to start the background section with the incidence of cancers in Uganda among young cancer survivors and the reproductive morbidity (consider merging current paragraph 2 and the note definition of reproductive morbidity).

**Response:** The background section has been revised and rearranged to convey a concise message. [see page background section, pages 3-5]

5. **Comment:** Purpose statement: The specific objectives are repetitive with what is already stated in the paragraph – consider merging all this information into one paragraph.

**Response:** The purpose statement and specific objectives have been revised and merged into one paragraph in the background. [see background, paragraph 5]

6. **Comment:** Methods Survey Approach:
   Setting: This paragraph is very long; suggestion to shorten the amount of detailed information on the Kyadondo County and its residents.

**Response:** Setting subsection in survey method has been revised. [see setting, under survey approach]

7. **Comment:** Eligibility Criteria: Suggestion to state the inclusion and exclusion criteria in sentence format versus a bulleted list. Please also include your rationale for only including cancer patients diagnosed from ages 0 to 25 years. The National Cancer Institute definition of adolescent and young adult cancer patient includes ages 15 to 39 years. Please explain why you are not including cancer patients aged 26 to 39 years.

**Response:** Formatted and revised eligibility criteria. Included rationale for choosing 0-25 years. Although, there is significant variation between societies for appropriate age defining young adulthood, the Society for Adolescent Health and Medicine defines young adulthood as a period ranging 18-25 years of age [16]. We assume among others [4, 16, 17], that this transition represents youth [18] and is associated with several unique physiological, social, and psychological phenomena that render them vulnerable to receiving inadequate health care. In addition, for many within in this age range, their reproductive expectations are not concrete as such they are a target group for reproductive survivorship compared to individuals 26-39 years of age. [see page 6, Eligibility section lines 5-16]

8. **Comment:** Sampling: This section is not clear; please consider revising for clarity.

**Response:** Revised to concisely describe how we arrived at the sample size for the survey. [see page 7, sample size section]

9. **Comment:** Recruitment strategy: Please explain how the message with the survey will be sent to potential subjects prior to the first call. Will information be mailed?

**Response:** A text message containing summarised information about the ongoing survey will be sent to the registered contacts of all potential subjects before the first call by the study team. [see page 8, under recruitment strategy]

10. **Comment:** Survey data collection, management, and analysis: The paragraphs describing the survey instruments and data collection are quite lengthy and somewhat repetitive. Consider revising to be more concise. For the data collection processes, please provide a rationale for making 10 attempts and up to 2 per day for the first contact.

**Response:** Survey data collection, management, and analysis revised and rearranged to a more concise version. To define a failed contact attempt, the investigators agreed that 10 call attempts made over 5 working days with at least two calls per day (morning and afternoon), was exhaustive considering available resources and would be the desired criterion. [see page 8-9, Survey data collection, management, and analysis section]

11. **Comment:** Methods Qualitative Approach:
    Setting: This paragraph is too long; suggestion to shorten the amount of detailed information in this paragraph.

**Response:** Setting for qualitative approach revised and formatted. [see page 10, under setting section]

12. **Comment:** Eligibility Criteria: Suggestion to state the inclusion and exclusion criteria in sentence format versus a bulleted list.

**Response:** Formatted eligibility section. [see page 10, under eligibility section]

13. **Comment:** In-depth interview instruments: Please move the sentences describing the interview guides located in the “Data collection process” paragraph into this section to provide more details regarding the semi-structured interview questions. Data collection process: This section can be shortened for better flow and clarity.

**Response:** The sections In-depth interview instruments and Data collection process have been revised and rearranged to convey a concise message. [see page 10-11]

14. **Comment:** Qualitative data management and analysis: Please provide more details on how many individuals will be coding the qualitative transcripts and how consensus between coders will be reached.

**Response:** Each transcript will be coded thrice, by a team of three investigators. To reach inter-coder consensus we shall use methods described by MA Cascio, et al. [27]. All axial codes and their representative open codes for all coders will be tabled and compared to identify discrepancies at the level of the axial codes. The axial codes are then sorted into overlapping and exact match categories, any residual points of non-consensus are discussed until unanimous resolution is reached. [ see page 11-12, under section Qualitative data collection, management, and analysis]

15. **Comment:** Trustworthiness: Consider removing this section from the manuscript.

**Response:** This section was deleted.

16. **Comment**: Discussion: Consider revising the last three paragraphs into one study strengths and limitations paragraph.

**Response:** The discussion has been revised and rearranged. [see page 13, under discussion]

**REVIEWER 2**

1. **Comment:** Have the authors described where all data underlying the findings will be made available when the study is complete? The [PLOS Data policy](https://journals.plos.org/plosone/s/materials-and-software-sharing) requires authors to make all data underlying the findings described in their manuscript fully available without restriction, with rare exception, at the time of publication. The data should be provided as part of the manuscript or its supporting information or deposited to a public repository. For example, in addition to summary statistics, the data points behind means, medians and variance measures should be available. If there are restrictions on publicly sharing data—e.g., participant privacy or use of data from a third party—those must be specified. Reviewer #2: No

**Response:** The survey questionnaires, abstraction forms and interview guides are included in the supporting information files. [see page 14, Availability of data and materials]

2. **Comment:** My first concern borders on the sampling method for the cross-sectional arm of the study - Will the authors recruit only the calculated sample size of 362 from the identified study population, or do they plan to recruit all consenting eligible survivors? If the former is the case, then the authors should calculate and adjust for non-response, define as well how they will select the sample population from the study population

**Response:** We plan to recruit all consenting eligible survivors. 362 is the only the minimum sample required to answer the objective.

3. **Comment:** My second concern relates to the questionnaire - was it adapted to the local population

**Response:** The survey questionnaire was adapted to the local consent and pre-tested before rolling out.

4. **Comment:** Thirdly, is there any compensation for the participants for their time?

**Response:** There is a compensation of 20,000 UGX for the survey and 40,000 UGX for the in-depth interview. [see page 8, paragraph 1, line 8 and page 12, paragraph, line 6]

5. **Comment:** Fourthly, the authors noted the possibility of adverse psychological effects by the study - How do they plan to monitor this? or do they have a plan for interim assessment of the magnitude of such psychological harm?

**Response:** The research assistants will be trained to observe emotional cues during the telephone survey and face to face in-depth interviews. In addition, the participants will have an option decline answering questions that are sensitive or halt the interview if they have overwhelming emotional fall out. In case of emotional fall out the participant will be referred to the study counsellor for further care. There will be interim assessment of the magnitude of such psychological harm. [see page 9, paragraph 1, line 3-6]

6. **Comment:** Finally, the authors should be specific on how the study will influence policy and practice.

**Response:** Results from this study will provide a foundation for awareness and advocacy for fertility preservation in at risk young cancer patients in Uganda. In addition, the study will provide valuable insight and justification for incorporating reproductive survivorship into the national cancer control plans. [see page 14, conclusion section]

Thank you so much. Look forward to the feedback.

Kind regards

Anthony Kayiira

Corresponding author
