# Supplementary material for: Understanding Reproductive Health among Survivors of Paediatric and Young adults (URHSPY) cancers in Uganda: A mixed method study protocol
Source: PLoS One. 2023 Apr 25;18(4):e0284969. doi: 10.1371/journal.pone.0284969 (PMC10128918; doi:10.1371/journal.pone.0284969)
Supplement: Attachment — Submitted filename: Response to reviewers .docx [file pone.0284969.s005.docx]

Friday, March 17, 2023

**Editor-in-Chief**

**PLOS ONE journal**

**RE: Response to reviewers for protocol # PONE-D-22-20391; Understanding Reproductive Health among Survivors of Pediatric and Young adults (URHSPY) Cancers in Uganda: a mixed method study protocol**
Thank you for accepting to review our manuscript. We have prepared a point-by-point response to the reviewers concerns in the body on this letter. Changes in the manuscript are tracked, and where needed highlighted in red.

**Journal requirements**
**Comment:** Please review your reference list to ensure that it is complete and correct. If you have cited papers that have been retracted, please include the rationale for doing so in the manuscript text or remove these references and replace them with relevant current references. Any changes to the reference list should be mentioned in the rebuttal letter that accompanies your revised manuscript. If you need to cite a retracted article, indicate the article’s retracted status in the References list and also include a citation and full reference for the retraction notice.

**Response:** The reference list has been reviewed and found to have no retracted papers. Any changes to the number references, are in line with changes to the manuscript text. i.e., removing or adding reference citations.

**REVIEWER 1**

**Comment:** The authors response to reviewers’ comments was thorough and addressed the primary concerns to strengthen their study protocol manuscript. The results of this study will provide novel information about the reproductive health climate of pediatric and young adult cancer patients living in Uganda. The revised manuscript is a significant improvement from the previous version. Since PLOS ONE does not copyedit accepted manuscripts, a suggestion is to conduct a thorough copy edit of the manuscript to correct for double words, spelling, grammar, and punctuation throughout the manuscript. For example, in the Background section, first paragraph, fifth sentence, “Incidence” should not be capitalized and in the 6th sentence, “Nonetheless” is listed twice. Another example is in the Discussion section, second paragraph, second sentence, “CATI based surgery” should be “CATI based survey”.

**Response:** Thank you for your comment. Extensive efforts have been made to edit grammar, punctuation and spelling throughout the manuscript. The paragraphs have also been reordered and revised to succinctly convey the message of the manuscript.

**REVIEWER 3**

**Comment:** By having experience and expertise in qualitative methodology as additional reviewer my focus has caught some additional points that would benefit from being addressed. My biggest concern is that by reading your protocol you have neither convinced me that the study has a mixed methods design, nor that Grounded Theory is adequately planned to be used. It seems more like two different studies are planned, if not you need to better reflect why this is a mixed methods study and have data being generated will be analyzed together and provide added value to each other. Grounded Theory is a complex method to use, and I cannot see that the objective or the description of the methodology is in line with how the method should be used. Some choices needs to be made regarding these issues, and then it must be described with consistency.

**Response:** Thank you for your comment. We have made necessary edits and additions to the overall protocol to reflect the explanatory sequential mixed method design and an elaborative description of the grounded theory methodology. Please see the sections; aims and objective under background, study design under method, and qualitative phase.

**Comment:** Keywords in abstract should be ordered alphabetically.

**Response:** Keywords ordered alphabetically.

**Comment:** Page 6 “Eligibility criteria”, I would suggest you to reconsider the age defining “young adults”, 18-39 would be more appropriate. See reference from The National cancer institute in the USA, They define young adults between 15-39, and from legal motives it may be simpler to include those from 18. “The national Cancer Institute. Adolescents and Young Adults with Cancer. Available online: <https://www.cancer.gov/types/aya>

**Response:** Thank you for your comment. We do acknowledge the age category for young adults outlined by the NCI is 15-39yrs. We chose 0-25 to encompass childhood and young adult cancer survivors, using a young adult definition of 18-25 years. We also acknowledge there is significant variation between societies for appropriate age definition of young adulthood, the Society for Adolescent Health and Medicine defines young adulthood as a period ranging 18-25 years of age [14]. We assume among others [3, 14, 15], that this transition represents youth [16] and is associated with several unique physiological, social, and psychological phenomena that render them vulnerable to receiving inadequate health care. These psychosocial factors are less likely to manifest among individuals >25 but <40 years.

Our main assumption is that young adults older than 25 years at diagnosis in our population are likely to have already had a child (this is based on national demographic health survey data). Considering our study is aimed at children and young adults who fall into the category of reproductive vulnerability i.e. likely not to have a family and likely to have unrealized reproductive expectations, we excluded individuals diagnosed with cancer older than 25 years.

**Comment:** Page 6 heading “Sample size”, incomplete sentence; In this study, the proportion of survivors that had or sired a pregnancy…”

**Response:** Necessary edits made. See sample size sub-section, line 3.

**Comment:** Not convinced that what you are doing is Grounded Theory, a form of mixed methods: Yes, but the qualitative plan could more easily be understood if you would keep it o have a qualitative approach where interviews are planned to be analyzed using a thematic method. If you choose to keep GT as the preferred method, it must be clear if you are supposed to develop theory, or study the processes in relation to the subject – which presently is NOT a part of the described objective, and you are not presenting any adequate reference to the method of GT.

**Response:** Thank you for your comment. We have made necessary edits and additions to the overall protocol to reflect the explanatory sequential mixed method design and an elaborative description of the grounded theory methodology. Please see the sections; aims and objective under background, study design under method, and qualitative phase.

**Comment:** Page 10; “In depth interview instruments”: Preferably I would like to see you interview guide, the sample questions suggest that those being interviewed are supposed to be those that have suffered from cancer – but from the objective, they are supposed to be guardians – unclear, please revise. (For some reason i cannot access supplements)

**Response:** Thank you for the comment. This an edit error. We have revised and removed the sample questions. Instead, we have included them in the supplement interview guides. (have attached these to the revision).

**Comment:** Doing qualitative interviews without being a researcher trained in the methods is not suitable – especially when using GT, the interviewer must have training in GT in order to make interviews that follows the process of GT. And also the same if it is supposed to be narrative interviews analyzed with thematic analysis

**Response:** Thank you for the comment. The first author is research fellow undertaking qualitative methodology. Significant time has spanned from the time of the initial protocol submission. During this time the author has gone through rigorous training in grounded theory and analysis. The third author has completed her PhD thesis and with considerable expertise in grounded theory methodology.

**Comment:** Similarly, regarding the “mixed methods design”, here in the protocol it is ok, but in the resulting article you must argue how the data analysis and presentation will be done in line with how it is expected to be done in mixed design. No adequate reference for mixed methods designs is present. My suggestion is that when you are planning to publish, that you separate it into two articles, since the mixed design is weak and the objectives of the two parts differentiate too much.

**Response:** We have made efforts to revise the protocol to best suit mixed method design, including furnishing with appropriate references. Our hope was that we perform a situation analysis with a population that has had a longer exposure time for the outcome of interest. Unfortunately, the long exposure time negates recall of circumstances surrounding treatment especially in survivors who suffered cancer at ages less than 18 years. The best population for the qualitative part would be current patients going through the cancer treatment. In this case parents being the decision makers for minors. If the data collected from both methods cannot be integrated, we welcome the idea of publishing separate manuscripts.

Thank you so much. Looking forward to the feedback.

Kind regards

Anthony Kayiira

Corresponding author
